# Simulating developmental diversity: Impact of neural stochasticity on atypical flexibility and hierarchy

**DOI:** 10.3389/fpsyt.2023.1080668

**Published:** 2023-03-15

**Authors:** Takafumi Soda, Ahmadreza Ahmadi, Jun Tani, Manabu Honda, Takashi Hanakawa, Yuichi Yamashita

**Affiliations:** ^1^Department of Information Medicine, National Institute of Neuroscience, National Center of Neurology and Psychiatry, Kodaira, Japan; ^2^Department of NCNP Brain Physiology and Pathology, Graduate School of Medical and Dental Sciences, Tokyo Medical and Dental University, Tokyo, Japan; ^3^Geobotica, Brisbane, QLD, Australia; ^4^Cognitive Neurorobotics Research Unit, Okinawa Institute of Science and Technology Graduate University, Okinawa, Japan; ^5^Integrated Neuroanatomy and Neuroimaging, Kyoto University Graduate School of Medicine, Kyoto, Japan

**Keywords:** autism spectrum disorder (ASD), computational psychiatry, predictive coding, flexibility, representation learning, neural noise, Bayesian brain, neural network

## Abstract

**Introduction:**

Investigating the pathological mechanisms of developmental disorders is a challenge because the symptoms are a result of complex and dynamic factors such as neural networks, cognitive behavior, environment, and developmental learning. Recently, computational methods have started to provide a unified framework for understanding developmental disorders, enabling us to describe the interactions among those multiple factors underlying symptoms. However, this approach is still limited because most studies to date have focused on cross-sectional task performance and lacked the perspectives of developmental learning. Here, we proposed a new research method for understanding the mechanisms of the acquisition and its failures in hierarchical Bayesian representations using a state-of-the-art computational model, referred to as in silico neurodevelopment framework for atypical representation learning.

**Methods:**

Simple simulation experiments were conducted using the proposed framework to examine whether manipulating the neural stochasticity and noise levels in external environments during the learning process can lead to the altered acquisition of hierarchical Bayesian representation and reduced flexibility.

**Results:**

Networks with normal neural stochasticity acquired hierarchical representations that reflected the underlying probabilistic structures in the environment, including higher-order representation, and exhibited good behavioral and cognitive flexibility. When the neural stochasticity was high during learning, top-down generation using higher-order representation became atypical, although the flexibility did not differ from that of the normal stochasticity settings. However, when the neural stochasticity was low in the learning process, the networks demonstrated reduced flexibility and altered hierarchical representation. Notably, this altered acquisition of higher-order representation and flexibility was ameliorated by increasing the level of noises in external stimuli.

**Discussion:**

These results demonstrated that the proposed method assists in modeling developmental disorders by bridging between multiple factors, such as the inherent characteristics of neural dynamics, acquisitions of hierarchical representation, flexible behavior, and external environment.

## 1. Introduction

Developmental disorders, such as autism spectrum disorders (ASDs), represent various symptoms involving perceptual, behavioral, cognitive, and social dysfunctions, and elucidating their pathological mechanisms is a challenging task. A fundamental difficulty in understanding developmental disorders is the fact that their symptoms are the results of complex and dynamic processes involving multiple factors, including neural systems, cognitive behavior, environment, and development learning. At the levels of cognition and behavior, in addition to their symptoms, people with ASD were reported to show reduced performance in a wide range of cognitive and behavioral tasks (1–4). At the level of the neural system, there are many findings related to the pathology of ASD, such as imbalance of neural excitations and inhibitions (5), altered variability in neural dynamics (6, 7), alterations in alpha oscillations (8), and abnormalities in subcortical areas including frontolimbic circuit, brainstem including superior colliculus, and autonomic nervous system (9–15). At the external environment level, it has been known that cognitive-behavioral interventions, such as structuring the environment and reducing stimulus ambiguity, alleviate symptoms of ASD (16, 17). However, despite the accumulation of these findings, existing theories of atypical development remain fragmentary because the target symptoms and the levels of explanations for each of these findings are different (18).

To address this issue, computational study has been expected to play a key role (18–21). This is because computational models can provide explanations bridging multiple levels in complex dynamical systems of the brain through quantitative simulations of the processes of neural, cognitive, and behavioral interactions that are difficult to observe and manipulate in actual biological systems.

One of the promising computational theories for developmental disorders is Bayesian brain hypothesis (22), also referred to as predictive coding theory (23, 24), Bayesian cognitive modeling (25–27), and free energy principle (28). In Bayesian brain hypothesis, the brain is considered to have the hierarchical Bayesian model that reflects the probabilistic structures in environment, and a hierarchical and probabilistic predictive process enables adaptive cognition and behavior. From the aspect of Bayesian brain hypothesis, it is proposed that symptoms of ASD are failures in Bayesian inference and abnormal acquisition of a hierarchical Bayesian model. Furthermore, the Bayesian brain hypothesis argued that these failures in inference and acquisition result from circular interactions between external stimuli and the internal brain dynamics in short- and long-term timescales (29–32). However, most ASD studies using the Bayesian brain hypothesis have focused on cross-sectional (i.e., short-term) behavioral measures such as reasoning and decision making, and there have been few studies focusing on long-term effects of environmental interactions and the acquisition/developmental learning process. For example, some studies attempted to fit theoretically driven hierarchical Bayesian models to behavioral data, and group differences in estimated values of model parameters between healthy and atypical developmental groups were investigated (33–35). In those studies, because a hierarchical Bayesian model has been constructed by researchers a priori, the process of acquiring a hierarchical Bayesian representation has not been examined.

Artificial neural networks, one of the computational modeling methods for brain function (36–38), could help investigate the developmental learning process because neural network models acquired internal representation reflecting external environment through synapse updating (39–43). In particular, a hierarchical recurrent neural network (RNN) model (44–46) has been widely applied for modeling higher cognitive function in the brain because this model has high similarity to the hierarchical system of the brain and capacity to reproduce complex dynamics. In addition to typical development (47–49), some studies investigated developmental disorders (50–53) and schizophrenia (54) as failures in the hierarchical neural system using hierarchical RNNs, and examined behavioral phenotypes and its relations to representations acquired in neural networks. These studies, referred to as neurorobotics, are promising for psychiatric research because they investigated the acquisition process of higher-order representations based on realistic and multidimensional sensorimotor sequence with the interaction of physical environment using a humanoid robot driven by an RNN (50–52, 54).

Recently, a neural network model that combines the properties of a hierarchical Bayesian model and RNN, referred to as predictive-coding-inspired variational recurrent neural network (PV-RNN), has been proposed (55). PV-RNN can embed complex stochastic sensorimotor signals in neural dynamics as a hierarchical Bayesian model through the developmental learning process. Therefore, PV-RNN can be considered a powerful tool for investigating the Bayesian brain hypothesis. Indeed, PV-RNN was useful for modeling uncertainty estimations (55), goal-oriented behavior (56), sensory attenuation (57), and social interaction (58–60).

**Figure 1:**
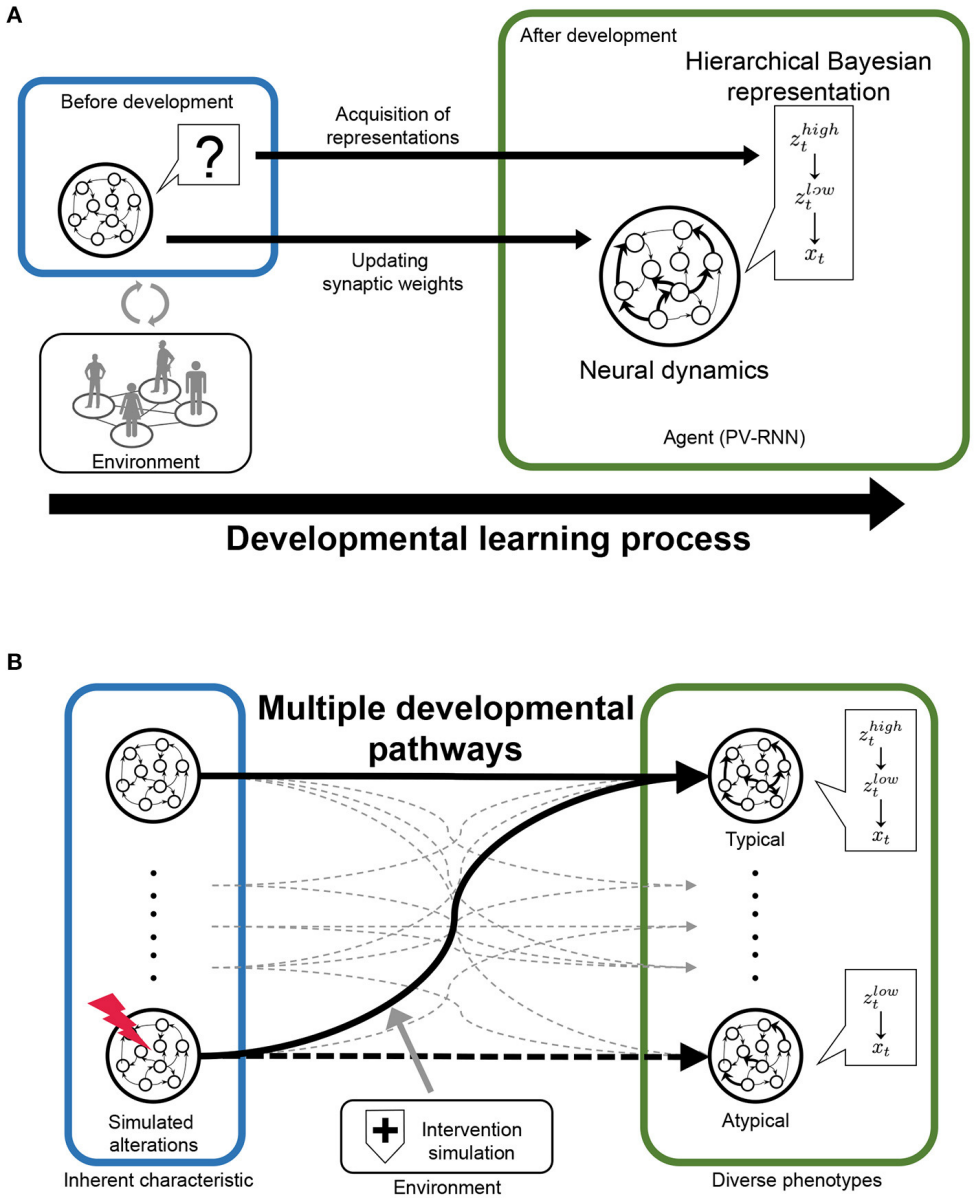
The scheme of “‘*in silico* neurodevelopment framework for atypical representation learning” proposed in this study. **(A)** The agent modeled by the hierarchical Bayesian neural network model (PV-RNN) must learn the hierarchical and probabilistic structure hidden in the observations in the developmental learning process. **(B)** The inherent characteristics of neural dynamics and environmental factors are simulated as experimental manipulation to understand divergence in the developmental process. *z*_*t*_ and *x*_*t*_ represent latent and observed variables, respectively.

In this study, we propose a novel and useful framework using PV-RNN for the understanding of typical and atypical developmental process, referred to as “*in silico* neurodevelopment framework for atypical representation learning” (Figure 1). The key point of the proposed framework is the integration of computational theory of hierarchical Bayesian models and neural network models as dynamical systems from the perspective of developmental learning. Specifically, in this framework, the developmental learning process of an agent is simulated in which the neural system acquires a hierarchical Bayesian representation in a self-organizing manner thorough interacting with the environment (Figure 1A). Furthermore, by manipulating the inherent characteristics of neural dynamics and environmental factors, this framework can reproduce the diversity in the developmental process, including typical and atypical development and possible interventions (Figure 1B). Namely, in the simulations, the environment generated observable signals based on the unobserved hierarchical and probabilistic generative process reflecting cognitive behavioral tasks. Through the developmental learning in this environment, the agent is needed to acquire hierarchical Bayesian models reflecting the environment structures under various conditions. After this process, the performance of the agent in the cognitive behavioral tasks and the effects of manipulations are evaluated. In these ways, the relationships between the inherent characteristics of neural dynamics, acquisitions of hierarchical Bayesian representation, behavioral phenotypes, and the effects of environmental factors including possible interventions can be quantitatively analyzed.

**Figure 2:**
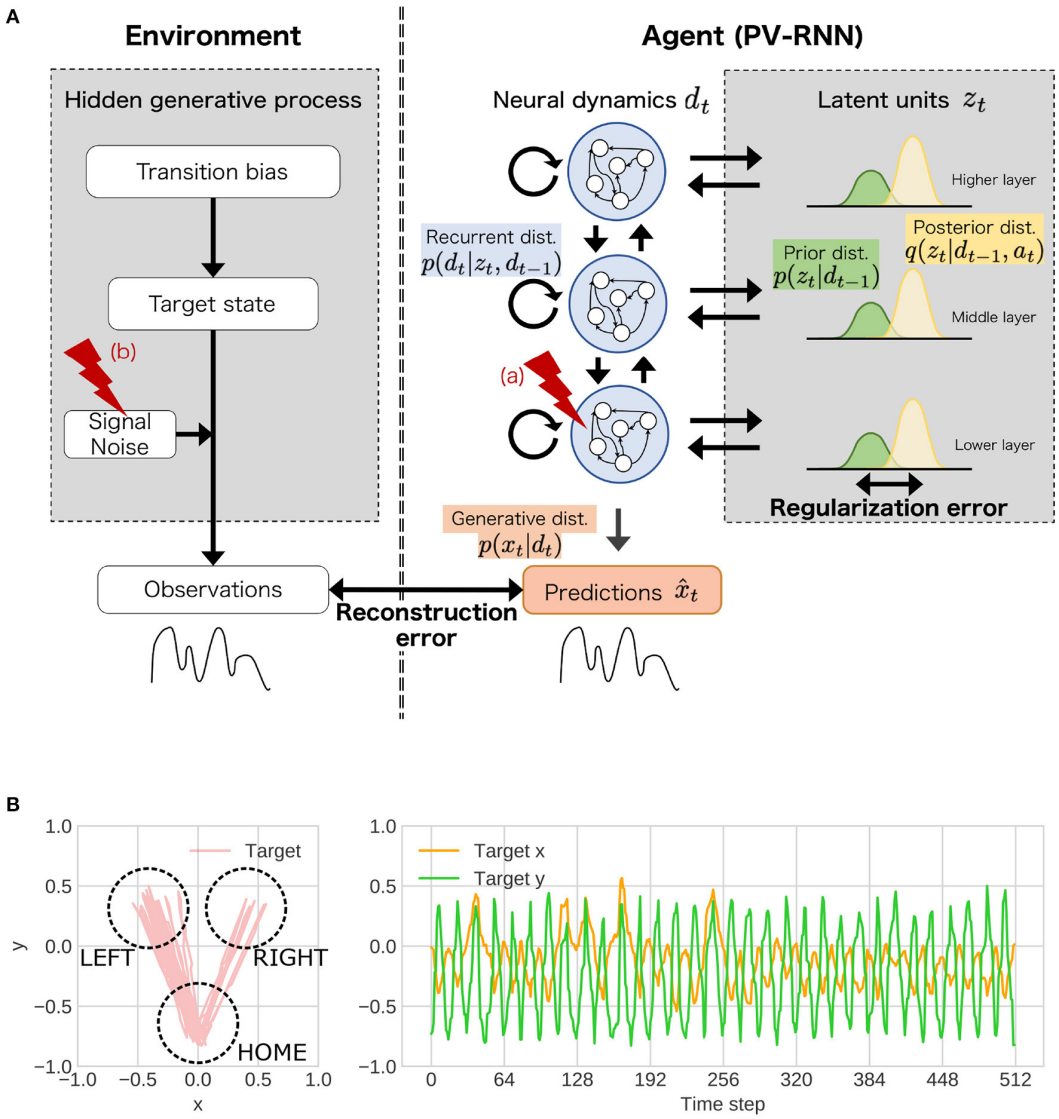
**(A)** The simulation experiments based on the proposed framework. In the experiments, as behavioral and cognitive task, flexibility task was used. To understand atypical developmental process, (a) the stochasticity in neural dynamics of lower layer, (b) noise level of observation signal was manipulated. *dist*. represents distribution. **(B)** An example of training sequences in the simulation experiments. These sequences repeated state transitions to LEFT or RIGHT (“target state”). The probability that the transition from HOME to LEFT is likely to occur is determined by “transition bias.” Transition bias was set to 0.76 (LEFT-biased sequences), and “signal noise” was set to low (stable environment condition) in the presented sequence. In the test phase, the transition bias switched at the middle point in the sequence to quantify flexibility.

As a proof of concept, we conducted a simulation experiment using the ‘*in silico* neurodevelopment framework for atypical representation learning framework (Figure 2). Specifically, we focused on the relationship between the acquisition of hierarchical and probabilistic representations reflecting environment structures and “reduced flexibility.” Indeed, reduced flexibility is one of the representative cognitive-behavioral phenotypes in ASD (2, 61, 62). Although many neural foundations related to reduced flexibility have been reported (2, 62), the mechanism between these neural alterations and the reduced inflexibility has not been well known. Therefore, in the simulations, we examined: (1) whether manipulating inherent characteristics of neural dynamics and external environment induces reduced flexibility; (2) whether these manipulations lead to the normal/abnormal acquisition of hierarchical Bayesian representations; (3) how the abnormalities in hierarchical Bayesian representation are related to reduced flexibility.

## 2. Materials and methods

### 2.1. Overview

The simulation experiments based on the proposed framework consisted of two components including an environment (left side in Figure 2A) and an agent (right side in Figure 2A). The environment generated observable signals following the unobserved hierarchical and probabilistic generative process, which is designed to measure flexibility. The agent was required to embed the covert hierarchical structures of environment into neural dynamics using only the observed signals through the developmental learning process. After the learning process, the ability of flexibility was tested in this environment. In the experiments, stochasticity in the neural networks (i.e., agent side) and noise level of the observation signals (i.e., environment side) in the learning process were manipulated as inherent characteristics and external environmental factor, respectively. Then, we investigated whether the changes in these factors impacted on the acquisition of hierarchical representations and flexibility.

### 2.2. Environmental stimuli and task setting

The observable signals were two-dimensional trajectories of objects that mimic reaching movements (Figure 2B) and were generated by three unobservable, hierarchical, and stochastic variables: “transition bias,” “target state,” and “signal noise” (left side of Figure 2A). Specifically, these sequences repeated the state transitions from HOME to LEFT or RIGHT (target) and return to HOME from LEFT or RIGHT. The transition bias represented the probability of the transition from HOME to LEFT, as a highest-order context in the environment. The target states (LEFT or RIGHT) were sampled from Bernoulli distributions parameterized by transition bias. The observable goal positions in one reaching movement were sampled from Gaussian distributions whose mean parameter corresponded to a central coordination of each target state and variance parameter corresponded to signal noise.

For the training, two sets of nine sequences (18 sequences in total) with 512 steps were generated with nine different transition probabilities (0.98, 0.87, 0.76, 0.65, 0.54, 0.43, 0.32, 0.21, and 0.10). Asymmetry of transition bias was used to improve the divergence of variances in the sequences. The agent learned to reproduce these sequences with diverse transition probabilities through the developmental learning process. In the test phase, the “flexibility” of the agent was tested using unknown test sequences whose transition bias was switched at the middle of the sequences. Namely, for the test sequences, two sequences with different transition biases (256 steps) were connected in which the transition bias in the second half of the test sequence was randomly sampled from the values in opposite directions to the transition bias of the first half.

The flexibility of the agent was evaluated in terms of the capability to perceive and follow change in the observations and unobservable context (i.e., transition bias) in these unknown test sequences. This quantification was inspired by flexibility tasks, such as the Wisconsin card sorting task (61), in which participants are required to detect changes of a rule or context throughout the task. The flexibility of the agent was evaluated by using two types of performance measures: 1) how accurately the network predicted observations (behavioral flexibility) and 2) how accurately the network inferred unobservable transition bias of the current sequence (cognitive flexibility). The details of the signal generation and quantification methods are shown in Supplementary Methods 1.1, 1.2, respectively.

The task settings presented here were designed to integrate motor control tasks and Wisconsin card sorting tasks. People with ASD have been reported to have alterations in sensorimotor processing (3, 4, 63), including the altered performance in the reaching movement task (3, 63). Based on these findings, observation signals in the current task were synthetically created to mimic reaching behavior, including seeing an object, predicting the movements of the object, and reaching the object. The observation signals in our task setting correspond to the moves of the target object, and the outputs of the neural network model correspond to visual and proprioceptive signals. In addition, the current task also includes a component of cognitive function measured by the Wisconsin card sorting tasks, i.e., flexibility. Indeed, individuals with ASD have been also reported to have reduced performance in the flexibility task (2, 61, 62). This component was implemented in the form that rules of object transitions (i.e., the transition bias) were switched without any notifications, and the agent needs to discover the switch.

### 2.3. Neurocognitive model

#### 2.3.1. Architecture of PV-RNN

The task for the agent was to acquire an internal representation that reflects the abovementioned hidden environment structure and flexibly adapt to unknown sequences. According to the Bayesian brain hypothesis, this problem for the agent can be described as follows. The agent constructs the statistical model *p*(*x*_≤ *T*_) = *p*(*x*_1_, *x*_2_, …, *x*_*T*_) approximating the true data distribution of the environment in which *x* and *T* represent the observed signals and length of sequences, respectively. The model of agent, PV-RNN (55), factorizes this distribution by introducing two latent variables, neural dynamic units *d*_*t*_ and probabilistic latent state units *z*_*t*_ (right side in Figure 2A).


$$\begin{array}{l} p\left(x_{\leq T}\right)=\int \cdot \cdot \cdot \int p\left(x_{\leq T},d_{\leq T},z_{\leq T}\right)dd_{\leq T}dz_{\leq T}\\ =\int \cdot \cdot \cdot \int p\left(x_{1}|d_{1}\right)p\left(d_{1}\right)p\left(z_{1}\right)\\ \overset{T}{\underset{t=2}{\prod }}p\left(x_{t}|d_{t}\right)p\left(d_{t}|d_{t-1},z_{t}\right)p\left(z_{t}|d_{t-1}\right)dd_{\leq T}dz_{\leq T} \end{array}$$

This equation indicates that the PV-RNN constructs *p*(*x*_≤ *T*_) using three components: prior distribution *p*(*z*_*t*_|*d*_*t*−1_), recurrent distribution *p*(*d*_*t*_|*d*_*t*−1_, *z*_*t*_), and generative distribution *p*(*x*_*t*_|*d*_*t*_). In addition, to estimate the latent states based on observations, approximate posterior (inference) distribution *q*(*z*_*t*_|*d*_*t*−1_, *a*_*t*_) was introduced. It should be noted that adaptive variables *a*_*t*_ are learnable parameters and save the error information about each training sequence. For the approximate posterior, the PV-RNN (55) uses *q*(*z*_*t*_|*d*_*t*−1_, *a*_*t*_), instead of *q*(*z*_*t*_|*d*_*t*−1_, *x*_*t*_) used in the variational recurrent neural network model (64). The use of *q*(*z*_*t*_|*d*_*t*−1_, *a*_*t*_) is inspired by the predictive coding theory (24), namely the posterior of latent states is inferred not directly based on external inputs *x*_*t*_, but based on prediction error.

**Figure 3:**
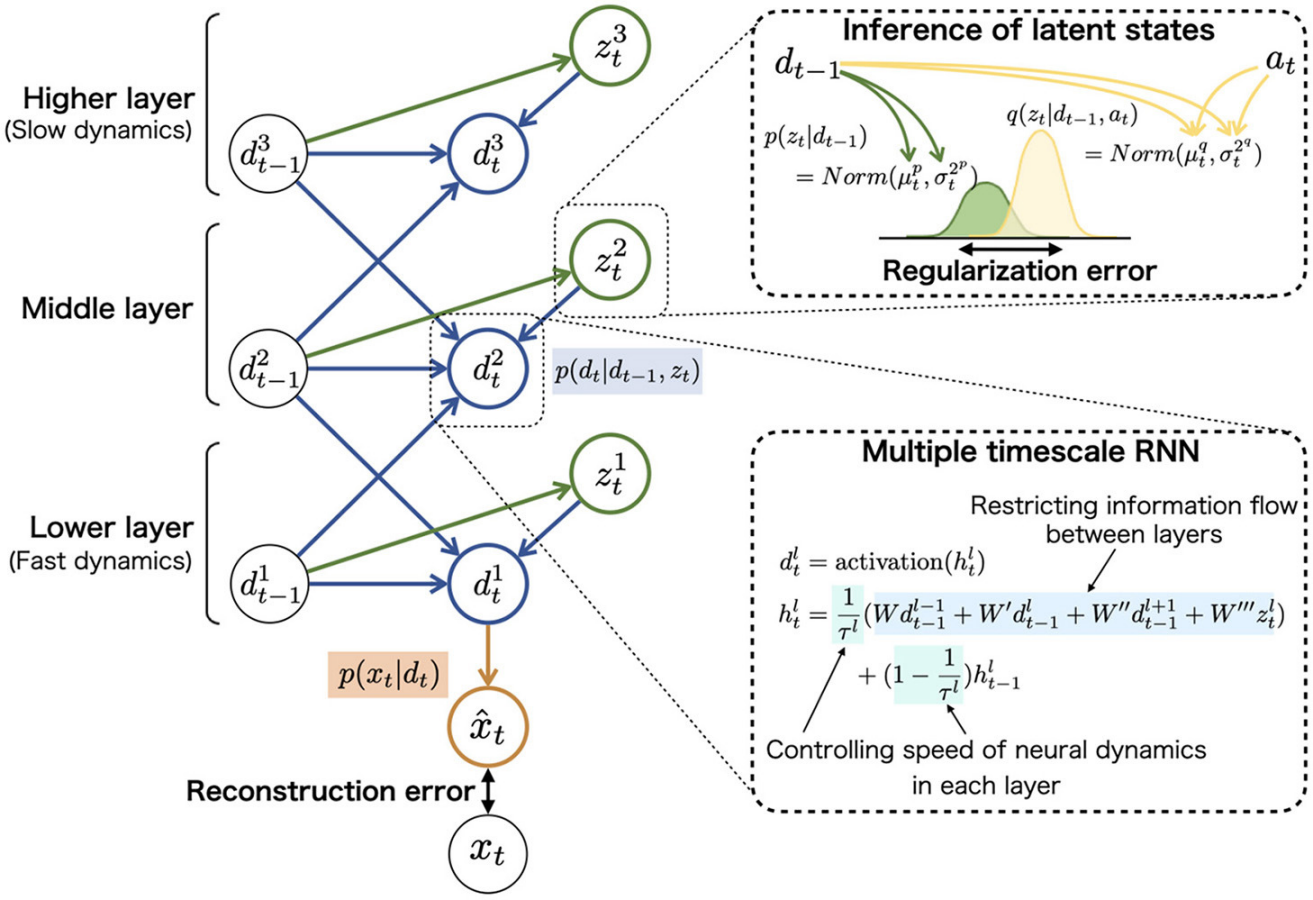
The graphical representation of PV-RNN architecture **(left)**. PV-RNN constructs hierarchical generation process in which the higher layer has larger time constant (slow neural dynamics) while the lower layer has smaller time constant (fast neural dynamics), as shown in **(bottom-right)**. In **(top-right)**, the inference process of *z*_*t*_ is illustrated. The right superscripts of symbols (i.e., *p* and *q*) are used to distinguish prior and approximate posterior distributions. The inference of posterior latent units $z_{t}^{q}$ is performed by propagating the errors in the reverse direction of arrows and updating the adaptive variables *a*_*t*_. This figure is simplified to improve readability, and detailed and accurate information of PV-RNN is shown in Supplementary Methods 1.3, 1.4.

These probabilistic distributions of mapping from the inputs to outputs were implemented in neural network models and refined through the learning (update of synaptic weights). For example, prior distribution *p*(*z*_*t*_|*d*_*t*−1_) assumed to follow the Gaussian distribution was represented using the mean and variance units (top-right in Figure 3). The neural network model corresponding to prior distribution inferred the mean and variance of latent units *z*_*t*_ using neural dynamics of *d*_*t*_.

The neural network corresponding to recurrent distribution *p*(*d*_*t*_|*d*_*t*−1_, *z*_*t*_) has a key role in the top-down and bottom-up flows of information in a hierarchical network (bottom-right in Figure 3). It is well known that the brain has hierarchical properties such as differed intrinsic neural timescales and distinctive anatomical connections, and the hierarchy may contribute to the complex cognitive functions (65, 66). The hierarchical nature of the PV-RNN was implemented to imitate these biological findings by providing different time constants for each layer and restricting the connections between the higher and lower layer units [multiple timescale RNN: MTRNN (46, 54)]. In addition, prior distribution *p*(*z*_*t*_|*d*_*t*−1_) and posterior distribution *q*(*z*_*t*_|*d*_*t*−1_, *a*_*t*_) have similar restrictions of the connections between the layers. For example, *z*_*t*_ units in the higher layer are inferred only using *d*_*t*_ units in the higher layer. Considering this hierarchy, the data distribution *p*(*x*_≤ *T*_) constructed by PV-RNN is factorized as follows (left side in Figure 3):


$$\begin{array}{l} p(x_{\leq T})=\int ...\int p(x_{1}|d_{1}^{1})\prod_{l=1}^{L}p(d_{1}^{l})\prod_{l=1}^{L}p(z_{1}^{l})\\ \;\prod_{t=2}^{T}\{p(x_{t}|d_{t}^{1})\\ \;\{p(d_{t}^{1}|d_{t-1}^{1},d_{t-1}^{2},z_{t}^{1})p(d_{t}^{L}|d_{t-1}^{L-1},d_{t-1}^{L},z_{t}^{L})\\ \;\prod_{l=2}^{L-1}p(d_{t}^{l}|d_{t-1}^{l-1},d_{t-1}^{l},d_{t-1}^{l+1},z_{t}^{l})\}\\ \;\{\prod_{l=1}^{L}p(z_{t}^{l}|d_{t-1}^{l})\}\}dd_{\leq T}dz_{\leq T} \end{array}$$

In this study, the number of layers was set to three. The number of *d*_*t*_ neural units and *z*_*t*_ units were set to (20, 10, 10) and (2, 2, 2), respectively, with the time constant at (2, 8, 32). Because *d*_*t*_ was used as deterministic variables, the integral of *d*_*t*_ was omitted in the following. The detailed architecture and generative processes are provided in the Supplementary Method 1.3.

#### 2.3.2. Loss function in the learning and test phase

Updates of synaptic weights in the learning phase and inference of latent states in the test phase follow the unified principle of minimizing the loss function. In the learning phase, losses were minimized by iteratively updating the synaptic weights and adaptive variables *a*_*t*_. As a result of learning, PV-RNN was expected to acquire efficient mapping from observed sensorimotor signals to hierarchical Bayesian representations. On the other hand, during the test phase, inference of latent states in posterior distribution was performed through modification of the adaptive variables *a*_*t*_ based on minimizing of the losses with fixing synaptic weights, called “error regression” (49).

In mathematical terms, the model parameters, such as synaptic weights and adaptive variables, were adjusted to maximize the similarity between the statistical model *p*(*x*_≤ *T*_) and the true data distribution of the environment. This is achieved by minimizing the negative of marginal log likelihood −log*p*(*x*). Using variational inference (67),


$$\begin{array}{l} -\log p\left(x\right)\leq \underset{\text{Reconstruction}\;\text{errors}}{\underset{︸}{-\overset{T}{\underset{t=1}{\sum }}E_{q\left(z_{t}|d_{t-1},a_{t}\right)}\left[\log p\left(x_{t}|d_{t}\right)\right]}}\\ \;+\underset{\text{Regularization errors}}{\underset{︸}{\overset{T}{\underset{t=1}{\sum }}D_{KL}\left[q\left(z_{t}|d_{t-1},a_{t}\right)||p\left(z_{t}|d_{t-1}\right)\right]}}. \end{array}$$

The right-hand side in this inequality is called variational free energy, and its negative is equivalent to the evidence lower bound (55, 68, 69). The first term, also called the reconstruction errors, is the negative log likelihood and reflects the differences between the data observations and predictions generated by the model. The second term, in which *D*_*KL*_ represents Kullback-Leibuler divergence, reflects the similarity between the prior distribution and posterior distribution and was proposed to have a regularization role (55). In PV-RNN, the weighting factor *w*^*l*^ for each hierarchy *l* was introduced to control the similarity between the prior distribution and posterior distribution as follows:


$$\begin{array}{l} Loss=-\overset{T}{\underset{t=1}{\sum }}E_{q\left(z_{t}|d_{t-1},a_{t}\right)}\left[\log p\left(x_{t}|d_{t}^{1}\right)\right]\\ \;+\overset{T}{\underset{t=1}{\sum }}\overset{L}{\underset{l=1}{\sum }}w^{l}D_{KL}\left[q\left(z_{t}^{l}|d_{t-1}^{l},a_{t}^{l}\right)||p\left(z_{t}^{l}|d_{t-1}^{l}\right)\right]. \end{array}$$

The weighting factor *w*^*l*^, referred to as “meta-prior,” was considered to control the stochasticity of neural dynamics (Supplementary Figures S1, S2) through the developmental learning process (55). In the developmental learning process, the neural dynamics is stochastic when the meta-prior is weak, while that is deterministic when the meta-prior is strong. In the test phase, the meta-prior plays a role in controlling the impact of the prior on the posterior; That is, a high meta-prior in the test phase leads to a strong effect of the prior on the posterior, while a low meta-prior weakens the effect. It is noted that the effects of meta-prior differ in the learning and test phase because synaptic weights are fixed in the test phase, and only inferred latent units in the posterior were updated. All parameters of PV-RNN (the synaptic weight and adaptive variables *a*_*t*_) were optimized using backpropagation through time by minimizing the loss function. As an optimizer, Adam (70) was used. The detail of loss deviation is provided in Supplementary Method 1.4.

### 2.4. Simulations of diversity in neural development

We manipulated several parameters in the simulation of the learning phase to investigate the relationships between inherent characteristics of neural dynamics, hierarchical Bayesian representation, behavioral and cognitive flexibility, and external environmental factors. First, as the inherent characteristics of neural dynamics, the stochasticity of the network in the developmental learning process was manipulated; This was implemented by changing the value of the meta-prior that controls the balance of two terms (reconstruction errors and regularization errors) in the loss function. This manipulation was attempted based on the previous theoretical studies suggesting that the stochasticity of the network (high or low neural noise) contributes to autistic symptoms (71, 72). In fact, some non-invasive studies have reported that participants with ASD showed altered neural noise (6, 7, 73, 74). Based on these findings and hypotheses, we expected that autistic-like phenomena, i.e., reduced flexibility, would be observed under both weak (high stochasticity) and strong (low stochasticity) meta-prior conditions, and the reduced flexibility would be induced by an abnormality in acquired hierarchical Bayesian representation. As a specific simulation setting, the meta-prior in the lower layer was set to 0.1, 1.0, and 10 as the weak, normal, and strong meta-prior conditions, respectively; the meta-prior in other layers was set to 1.0.

The second manipulated parameter was the level of noises included in the environmental stimulus during the developmental learning process; This is motivated by the well-known observations that reducing ambiguity in stimulations and the structuring environment promotes learning and improve behavioral and cognitive functions in children with ASD (16, 17). The large noise condition and small noise condition were tested by changing the levels of signal noise corresponding to the changes in the ambiguity of the states (LEFT, RIGHT, and HOME). Based on the findings related to interventions for people with ASD (16, 17), we hypothesized that less flexibility and alterations in the hierarchical Bayesian representations would be observed under large noise condition (noisy environment) than small noise condition (stable environment).

### 2.5. Implementation and statistical analysis

Python and PyTorch (75) were used in the experimental simulation to generate training and test sequences and implement the neural network model. Both R (76) and Python were used for visualization and statistical analysis. The 20 networks were trained in each condition. In each analysis, values outside of 1.5 times the quantile range in each condition were removed as outliers. Therefore, the number of conditions was inconsistent in each analysis. To compare between meta-prior conditions, analyses of variance (ANOVA) between-subject were used (three levels, normal, strong, and weak meta-prior). The interaction effects of meta-prior and signal noise were analyzed using a three (meta-prior conditions) × two (stable and noisy environments) ANOVA. In *post-hoc* multiple comparison, Shaffer's modified sequentially rejective Bonferroni procedure was used.

## 3. Results

### 3.1. Behavioral and cognitive flexibility in hierarchical Bayesian RNN

**Figure 4:**
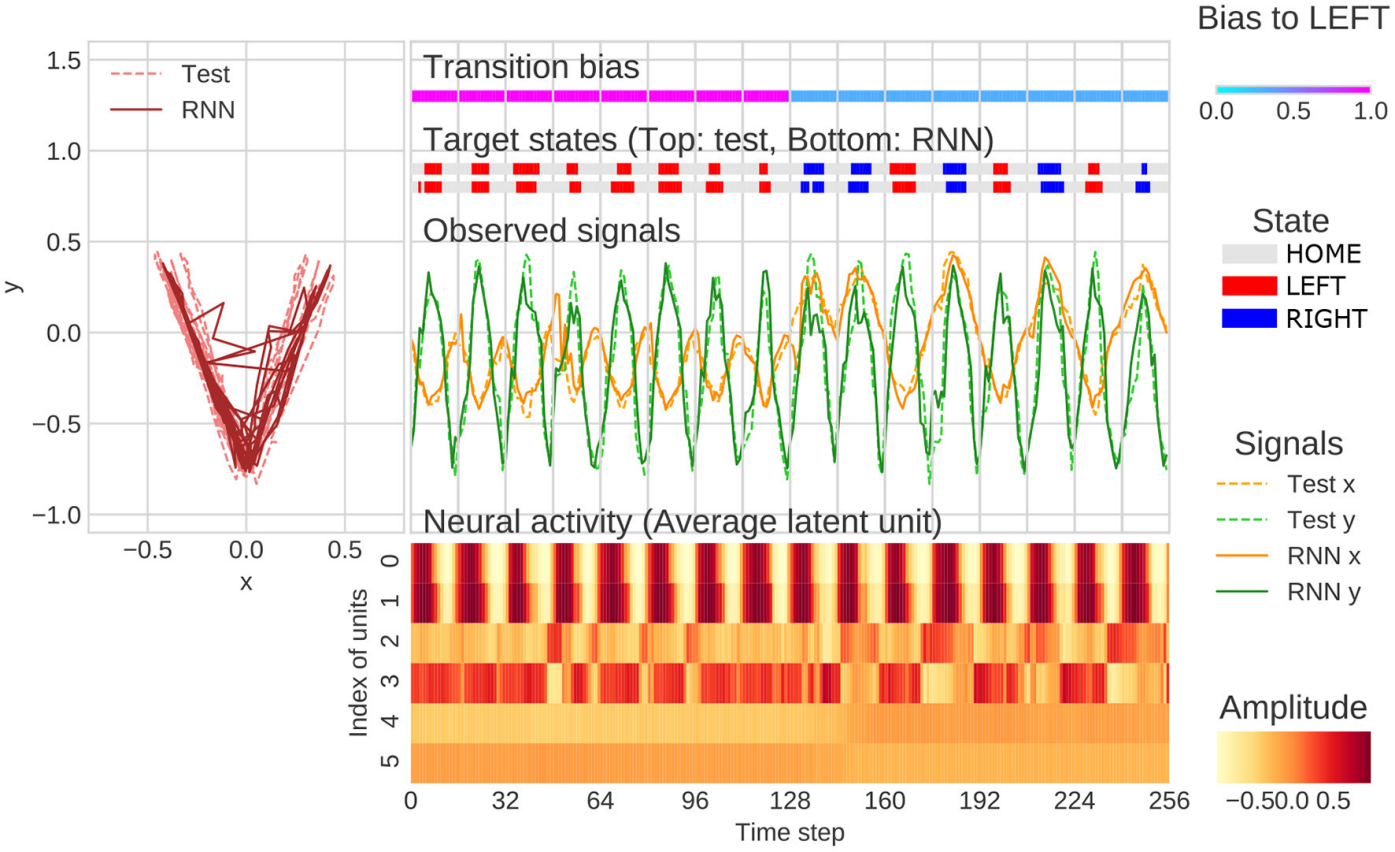
An example of flexibility tasks under normal meta-prior condition. In the top of figure, RNN generations and test sequence are plotted on two-dimensional plane **(left)** and along time axis **(right)**. The unit0 and unit1, unit2 and unit3, and unit4 and unit5 reflect the lower layer, middle layer, and higher layer, respectively. The latent units coding mean parameters of Gaussian distributions are plotted in the figure rather than *z*_*t*_ itself. In the figure, only the 128 steps before and after switching of the transition bias are plotted.

The representative example of generation of behavioral sequence and neural activities with the value of meta-prior referred to as the “normal meta-prior” condition was presented in Figure 4. The output sequences of RNN and test sequences were seemingly concordant not only at the observation signal (xy-coordinate) level, but also at the state transition level (i.e., HOME/LEFT/RIGHT). This indicated that the network successfully predicted unknown observations and adapted to the changes in the observation signals based on hierarchical internal representations acquired through the developmental learning.

Qualitative inspection indicated that the hierarchical representation of each latent unit played a different functional role. For example, the activities of latent units of the lower layer (unit0 and unit1) were synchronized with the y-axis in the behavioral trajectories. In the middle layer, unit2 was active when target states moved to RIGHT and unit3 was active in the opposite direction. The higher layer units, such as unit4 and unit5, appeared to be related to the probability of transitions to LEFT and RIGHT. Specifically, unit5 was active in generating LEFT-biased sequence (first half of Figure 4), and unit4 was active in generating RIGHT-biased sequence (last half of Figure 4). The distinct role of the middle layer and higher layer can be clearly observed in the last half of Figure 4. In this period, unit4 was continuously active because of RIGHT-biased generation even when LEFT-transition occurred (probabilistic effect on outputs). In contrast, unit3 was only active when LEFT-transition occurred (direct effects on target states). These observations indicated that the PV-RNN with normal meta-prior condition acquired hierarchical representation, which reflected the structures of environment and were flexible enough to adapt not only to the observable stimulus changes but also to the unobservable context switching.

**Figure 5:**
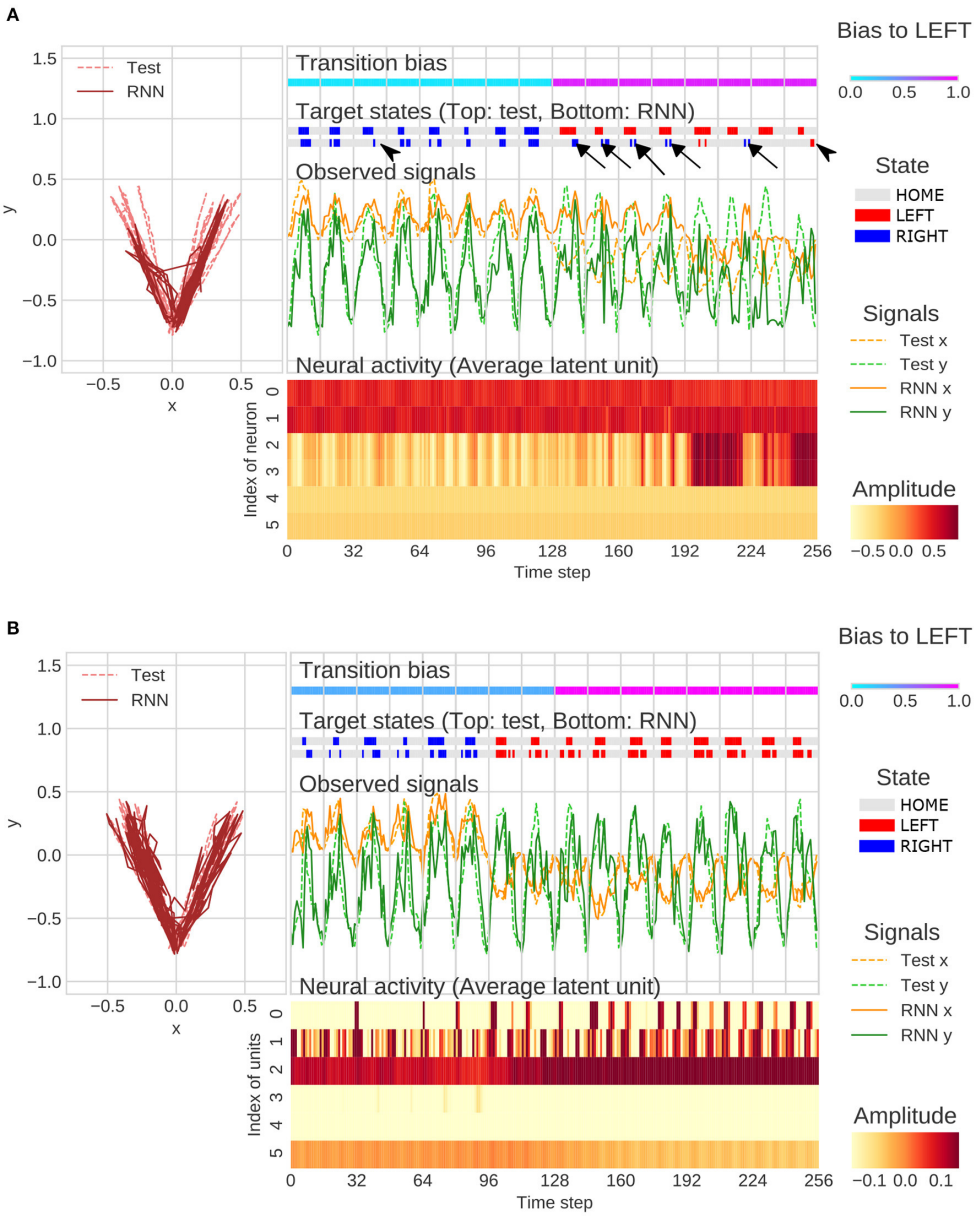
**(A)** Example of flexibility tasks under strong meta-prior condition. The arrows and arrowheads represent perseveration errors and timing mismatches, respectively. The latent units coding mean parameters of Gaussian distributions were plotted in figure rather than *z*_*t*_ itself. **(B)** Example of flexibility tasks under weak meta-prior condition. The range of color plot adjusted to activities of higher latent units although the max and min values in lower- and middle-units surpassed the ranges of those plotted. In the figures **(A, B)**, only the 128 steps before and after switching of the transition bias are plotted.

Under the strong meta-prior condition, the network failed to accurately predict the observations in the test phase. For example, movement timing of generated sequence did not match to a test sequence (arrowheads in Figure 5A). In addition, the network under strong meta-prior condition was unable to respond to the changes in context (probability of transitions) in the target states and repeated previous output patterns (perseveration errors; arrows in Figure 5A). Indeed, activities of higher layer units (unit4 and unit5) did not change at the point when transition bias switched in the test sequence. On the other hand, these failures in behavior including perseverative errors were not observed under the weak meta-prior condition (Figure 5B). However, neural activities seemed to be relatively noisy and unstable, and the functional roles of each layer of latent units were not clear compared to the normal meta-prior.

To confirm this qualitative evaluation, two types of measure were introduced: behavioral and cognitive flexibility. Behavioral flexibility was the ability to accurately adapt to observable signal changes and quantified using the percentage of the agreement between the states of observations and the states of predictions by the networks. On the other hand, cognitive flexibility was evaluated using the correlations between true values of transition bias in the test sequences and the activities of latent units in the higher layer of the networks. Therefore, cognitive flexibility reflects the efficacy of representation learning in terms of passive inference for higher-order context and the “insight” for changes of higher-order hidden context (transition bias) in the environment.

**Figure 6:**
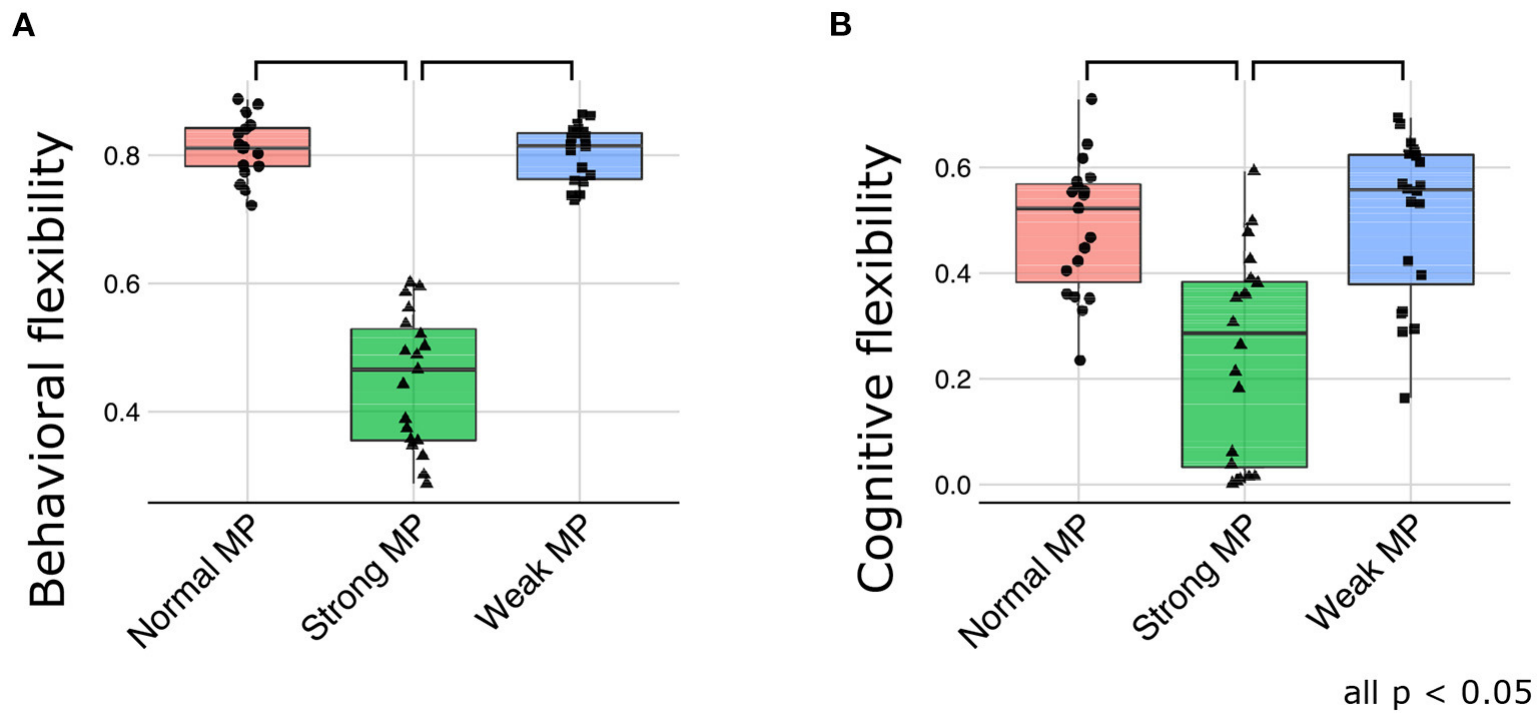
The quantitative evaluation about behavioral flexibility **(A)** and cognitive flexibility **(B)**. MP represents meta-prior.

Consistent with the qualitative evaluations, the behavioral flexibility was declined under strong meta-prior condition [*F*_(2, 51)_ = 152.5871; *p* < 0.0001 using ANOVA, and *t*_(51)_ = 15.0647; *p* < 0.0001 at normal > strong, and *t*_(51)_ = 14.9831; *p* < 0.0001 at weak > strong in *post-hoc* tests; Figure 6A]. Furthermore, cognitive flexibility declined more in strong meta-prior condition than weak and normal prior conditions [*F*_(2, 56)_ = 15.6619; *p* < 0.0001 using ANOVA, and *t*_(56)_ = 4.6497; *p* < 0.0001 at normal > strong, *t*_(56)_ = 5.0041; *p* < 0.0001 at weak > strong in *post-hoc* tests; Figure 6B].

### 3.2. Hierarchical and probabilistic representation for active generation

To further examine the functional role of the latent units in each layer of PV-RNN, we adapt deep learning technique called “latent space traversal (LST).” In the LST, the changes in the network predictions were investigated when the activity of single target latent unit was intentionally manipulated (77, 78). This makes it possible to functionally, causally, and operationally examine whether neural units code output information and to reject the possibility that the activity of higher layer units is passively responding to bottom-up signals. Therefore, the LST method focused on the decoding (active generation) ability while cognitive flexibility focused on the encoding ability (passive inference), although both were used for evaluation of representation learning.

The LST analysis was conducted as follows. One sequence of 1,024 time steps was generated by setting the activity of the target latent unit at a particular fixing value. This process was repeated by changing the fixing values ranging from –1.0 to 1.0. Properties of the generated sequences were evaluated in terms of the ratios of time steps staying with HOME and the number of LEFT transitions, and so on. Examples of generated sequences using LST were shown in Supplementary Figure S3.

**Figure 7:**
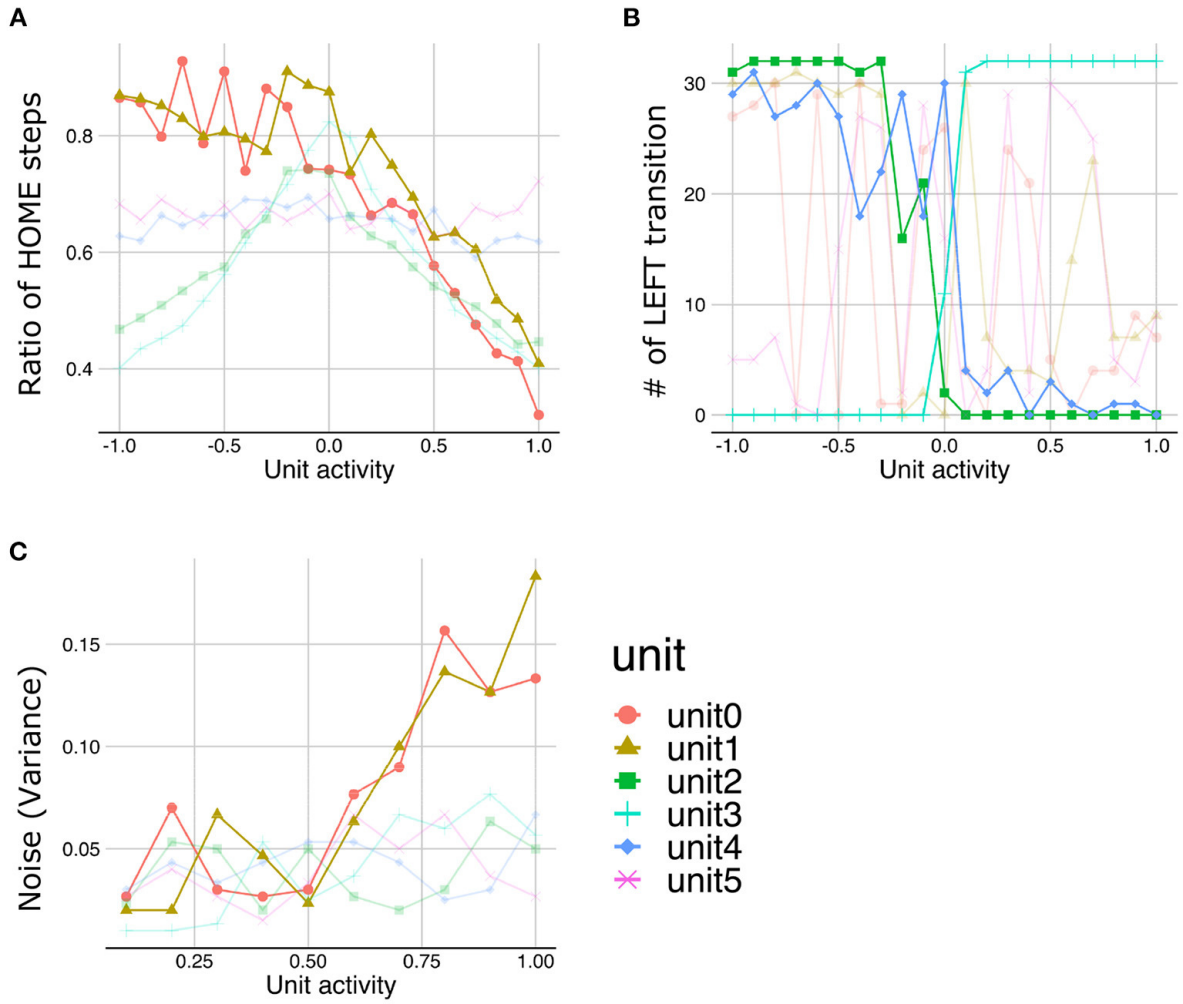
The results of latent space traversal under normal meta-prior condition. The properties of generated sequences (y-axis) changed depending on fixed activation values (x-axis) of one particular unit. Changes in the number of steps staying with HOME states **(A)** and the numbers of transition to LEFT states **(B)** were plotted. **(C)** Changes in the variances of generations were plotted when activities of units inferring variances of latent units were fixed. Irrelevant lines are plotted in a pale color to improve readability.

The LST under normal meta-prior condition demonstrated that the lower the activities in unit0 and unit1, the more time steps staying with HOME state (Figure 7A), suggesting that the lower layer units (unit0 and unit1) coded the y-axis movement. Similarly, the manipulations in the activities of middle layer units (unit2 and unit3) and higher layer units (unit4) lead the changes in the transition to the LEFT state, suggesting that these units coded LEFT/RIGHT transitions (Figure 7B). Note that the slope of the changes in the number of LEFT transitions induced by the higher layer unit manipulations is shallower than those induced by the middle layer unit manipulations. This observation suggests that the activity of the higher layer unit likely codes probabilistic information (i.e., transition bias), while the activities of middle layer units directly were associated with target state (i.e., LEFT or RIGHT) with an all-or-nothing manner. In addition, LST analysis applied to the variance units demonstrated that the variances of generated sequences increased as the activities of variance unit in the lower layers increased (Figure 7C). This unit seemed to code the amount of noise in the predicted signals (i.e., signal noise). These results suggested that the PV-RNN under the normal meta-prior condition could acquire hidden hierarchical and probabilistic structures of the environment in terms of not only passive inference but also active generation.

**Figure 8:**
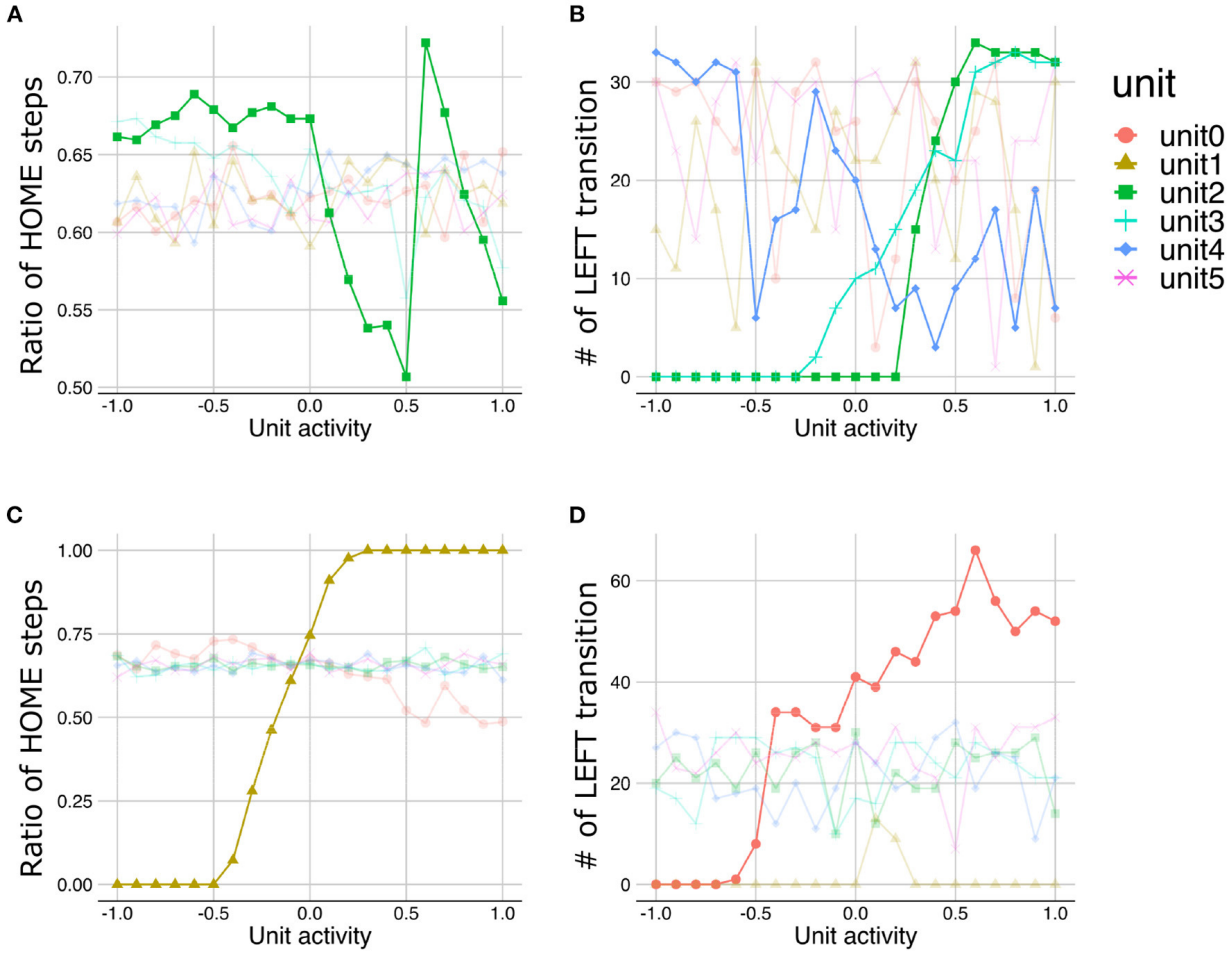
The results of latent space traversal under strong **(A, B)** and weak meta-prior condition **(C, D)**. Irrelevant lines are plotted in a pale color to improve readability.

The LST analysis with different meta-prior setting conditions demonstrated altered hierarchical representations. For example, under the strong meta-prior condition, lower layer units and higher layer units did not have distinct roles, and several levels of functions are intermingled in the middle layer. Namely, the activities of unit0 and unit1 (lower layer) did not have the effects on steps staying in HOME state and the number of LEFT transitions (Figures 8A, B). The activities of unit4 (higher layer) did not have clear effects on the sequence generations (i.e., association of activities and properties of generated sequences have several outliers) (Figure 8B). On the other hand, changes in unit2 and unit3 (middle layer) had effects on the time steps staying HOME state (Figure 8A) and in the LEFT transitions (Figure 8B). These observations suggested that under the strong meta-prior condition, the representations in each layer were not good, which was consistent with the observations of poor behavioral and cognitive flexibilities.

On the other hand, under the weak meta-prior condition, unit1 and unit0 had very clear effects on the y-axis movements (Figure 8C) and LEFT transition (Figure 8D), respectively. However, unit2, unit3 (middle layer), unit4, and unit5 (higher layer) had no effects on generated sequences. Therefore, under weak meta-prior condition, it seemed that latent representations in the lower layer were effective, but those in higher layer were ineffective.

To quantitatively confirm these findings, we defined a measure referred to as “generative hierarchy,” which represents the total amount of the causal effect of a network in terms of active generation. Namely, the latent units of the network have stronger causal effects for output sequences when the generative hierarchy of a network is high. The detailed procedure is as follows: first, in the LST analysis, correlations between the manipulated values of a particular latent unit (horizontal axis in Figures 7, 8) and behavioral properties of generated sequences including the number of transitions to each state, the number of stay steps in each state, and the variance in each state (i.e., the vertical axis in Figures 7, 8) were calculated. The maximum value of the correlations over all properties was calculated based on the assumption that this value represents the efficacy of each latent unit on behavioral generation. Finally, the average of the efficacy of the latent units in each layer was used as the generative hierarchy of each layer in one network.

**Figure 9:**
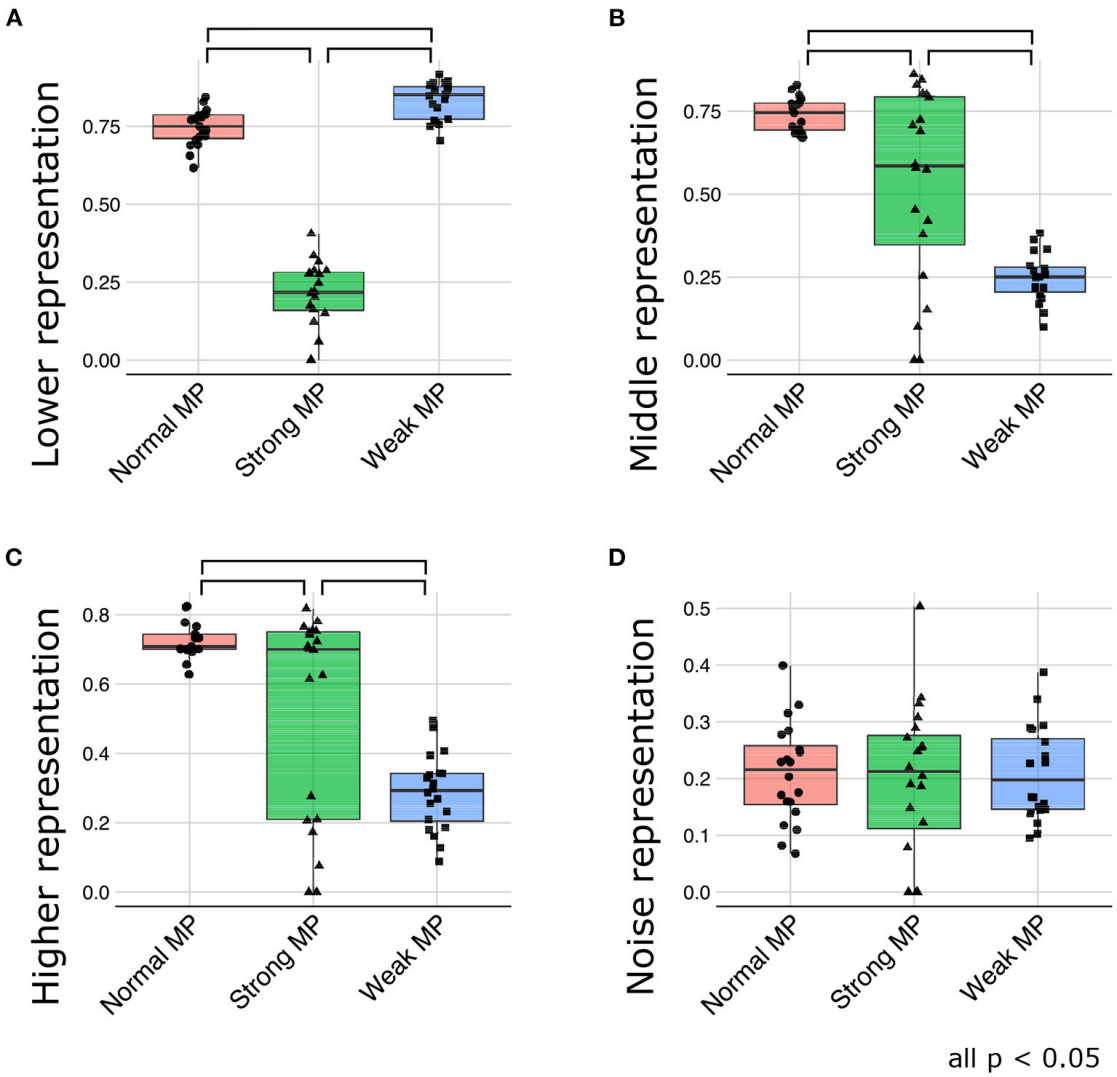
**(A–D)** The generative hierarchy under each condition. MP represents meta-prior.

Figure 9 depicts the generative hierarchy under each meta-prior condition. As expected, to sum up all the layers, generative hierarchy of the latent representations in normal meta-prior conditions seemed to be better than in other conditions. On the other hand, under weak meta-prior condition, the generative hierarchy in the middle and higher layer was poor, although that in the lower layer was comparable to normal meta-prior condition. The generative hierarchy under strong meta-prior condition was reduced in all layers compared to normal meta-prior condition except for noise representations of variance units.

These observations were confirmed by the following statistical analyses. The generative hierarchy was best under weak meta-prior condition in the lower layer [Figure 9A; *F*_(2, 56)_ = 361.8663; *p* < 0.0001, *t*_(56)_ = 3.4104; *p* = 0.0012 at weak > normal, *t*_(56)_ = 24.8918; *p* < 0.0001 at weak > strong, and *t*_(56)_ = 21.1603; *p* < 0.0001 at normal > strong]. However, in the middle layer (Figure 9B), generative hierarchy was the best in normal meta-prior condition than strong and weak meta-prior conditions [*F*_(2, 53)_ = 33.5184; *p* < 0.0001, *t*_(53)_ = 8.1367; *p* < 0.0001 at normal > weak, *t*_(53)_ = 3.5753; *p* = 0.0008 at normal > strong, *t*_(53)_ = 4.7977; *p* < 0.0001 at strong > weak]. Similar to the middle layer, in the higher layer (Figure 9C), the normal meta-prior condition showed the best generative hierarchy [*F*_(2, 54)_ = 24.3196; *p* < 0.0001, *t*_(54)_ = 6.9503; *p* < 0.0001 at normal > weak, *t*_(54)_ = 3.2721; *p* = 0.0019 at normal >strong, and *t*_(54)_ = 3.8371; *p* = 0.0003 at strong >weak]. The differences in the generative hierarchy of variance units were not significant [Figure 9D; *F*_(2, 57)_ = 0.0769; *p* = 0.9261].

These results suggested that the networks under a weak meta-prior condition generated using only lower layers; it did not have sufficient hierarchical and disentangled representations in term of active generation, and the hierarchical representations were effective only during passive inference. On the other hand, the networks under a strong meta-prior condition showed the abnormalities in hierarchical representations in terms of both active generation and passive inference.

### 3.3. The buffering effect of environment on representation learning

**Figure 10:**
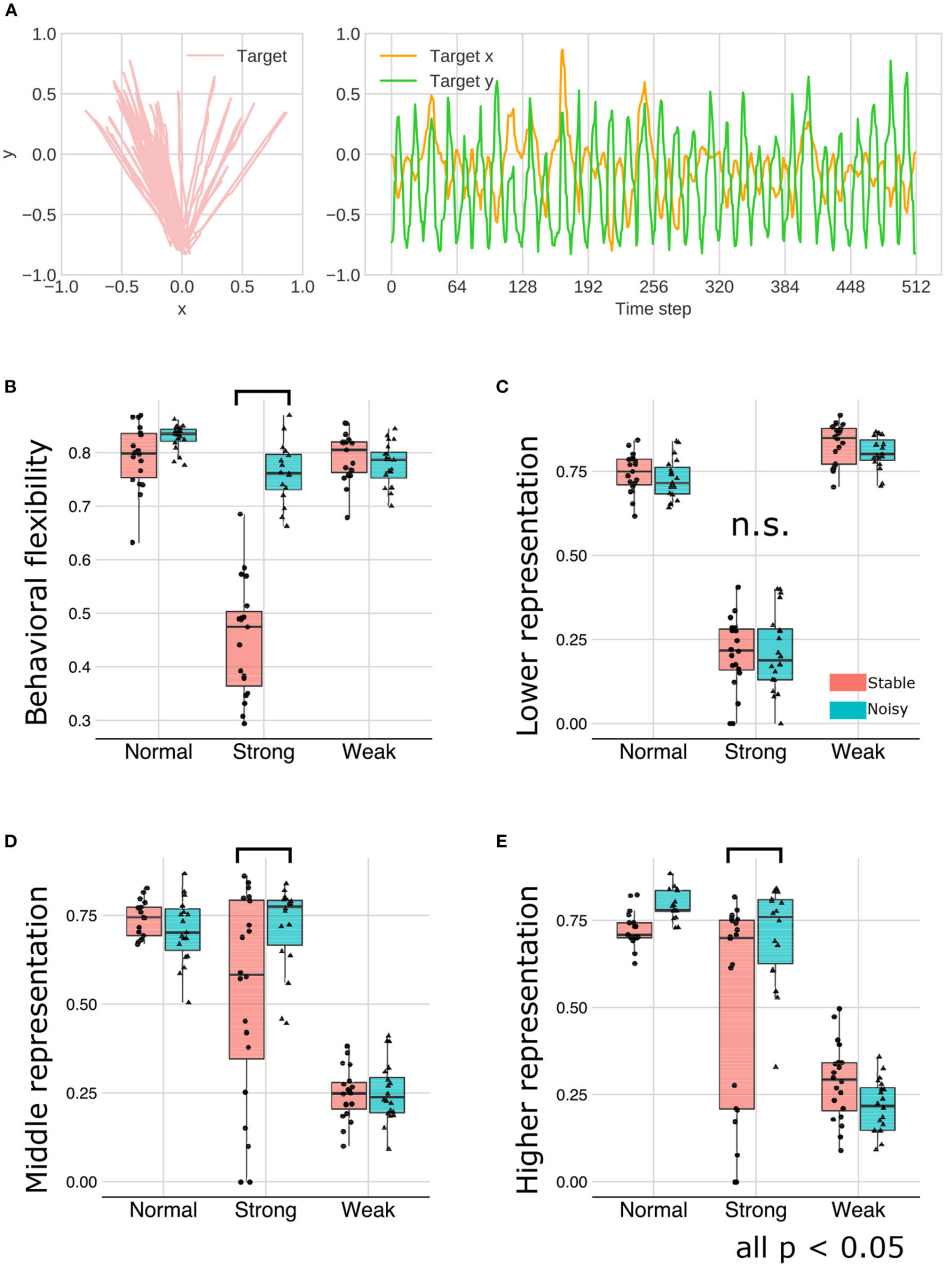
**(A)** The training sequence in noisy environment condition in which transition bias was set to 0.76 (LEFT-biased sequences). **(B–E)** The interaction effect between the environment and meta-prior. The results of statistical test were showed under only strong meta-prior condition.

As an external environmental factor during the developmental learning process, the noise level of the observation signals was manipulated. This experiment is motivated by the well-known phenomenon in education and support for children with ASD, namely that reducing ambiguity in stimulations and the structuring environment promote learning and improve behavioral and cognitive functions (16, 17). In this experiment, we manipulated the signal noise level included in the training and test sequences and examined the interaction effect between meta-prior and noise level on the representation learning. Figure 10A illustrated a representative example of behavioral sequence for training under the “noisy” environment condition in which LEFT and RIGHT states were not clearly distinguishable, in contrast to “stable” signal noise condition (Figure 2B).

As described earlier, strong meta-prior condition showed reduced flexibilities (behavioral and cognitive: Figure 6) and poor generative hierarchy (Figure 9). However, in the noisy environment condition, the behavioral flexibility under strong meta-prior conditions was improved (Figure 10B). Furthermore, the networks under strong meta-prior and noisy environment condition partly acquired the hierarchical representations, specifically in the middle (Figure 10D) and higher layer (Figure 10E). However, noisy environment under strong meta-prior condition did not induce the improvement of generative hierarchy in the lower layer (Figure 10C).

These observations were confirmed by the following statistical analyses. There were significant main effects of meta-prior and environment [*F*_(2, 105)_ = 116.4491; *p* < 0.0001 in meta-prior, *F*_(1, 105)_ = 90.8178, *p* < 0.0001 in environment]. In addition, the interaction effect between the environment and meta-prior on behavioral flexibility was significant [interaction effect *F*_(2, 105)_ = 72.7390, *p* < 0.0001]. Furthermore, the difference of behavioral flexibility between environment conditions was significant under strong meta-prior condition [the simple effect of environment on strong meta-prior condition *F*_(1, 105)_ = 222.8984; *p* < 0.0001]. However, the interaction effects on cognitive flexibility were not significant [*F*_(2, 112)_ = 1.7649; *p* = 0.1759], although main effects of meta-prior [*F*_(2, 112)_ = 28.7819; *p* < 0.0001] and noise level of environment [*F*_(1, 112)_ = 5.5784; *p* = 0.0199] were significant. Moreover, the strong meta-prior condition under noisy environment improved generative hierarchy in the middle layer [interaction effect *F*_(2, 107)_ = 6.3325; *p* = 0.0025, and simple effect of environment on strong meta-prior *F*_(1, 107)_ = 16.1836; *p* = 0.0001] and in the higher layer [interaction effect *F*_(2, 106)_ = 7.1059; *p* = 0.0013, and simple effect of environment on strong meta-prior *F*_(1, 106)_ = 14.5995; *p* = 0.0002]. The interaction effect between the environment and meta-prior on lower representations was not significant [*F*_(2, 111)_ = 0.3530, *p* = 0.7033].

Therefore, under the strong meta-prior condition, increased signal noise improved the behavioral flexibility and acquisition of the hierarchical Bayesian representation.

## 4. Discussion

In this study, we proposed a new research framework for understanding the pathological mechanisms of the atypical developmental process, using state-of-the-art computational model, PV-RNN. This framework comprehensively includes simulations of the multiple factors related to developmental disorders, for example, the neural dynamics, hierarchical Bayesian representation, cognitive-behavioral phenotypes, developmental learning processes, and the environment. In this framework, these factors could be manipulated without any restriction and analyzed quantitatively.

**Figure 11:**
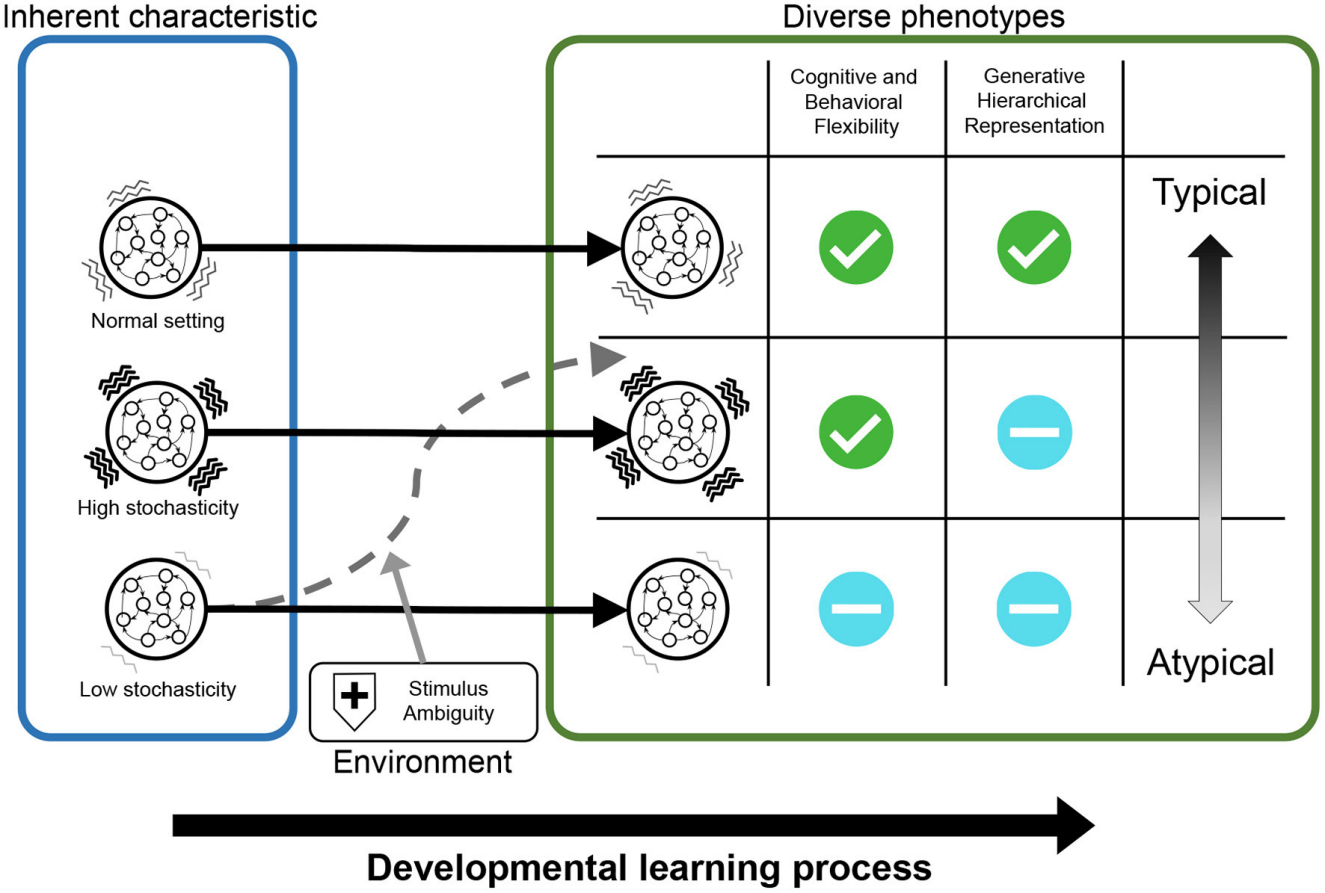
The results of simulation experiments were graphically summarized. The networks with normal neural stochasticity were able to acquire hierarchical representations, including higher-order representation, and exhibited good behavioral and cognitive flexibility. When the neural stochasticity was high in the learning process, top-down generation using higher-order representation (i.e., generative hierarchy) was impaired, although the flexibility did not differ from that of the normal settings. On the other hand, when the neural stochasticity was low in the learning process, the networks demonstrated reduced flexibility and abnormal hierarchical representation. However, this altered acquisition of higher-order representation and flexibility was ameliorated by increasing the level of noises in external stimuli.

As an example, in experiments using this framework, we analyzed the relationships between inherent characteristics of neural dynamics, hierarchical Bayesian representation, the properties of external stimulus, and inflexibility, which is cognitive-behavioral phenotype observed in patients with ASD. Particularly, this study investigated: (1) whether manipulating inherent characteristics of neural dynamics and external environment induces reduced flexibility; (2) whether these manipulations lead to the normal/abnormal acquisition of hierarchical Bayesian representations; and (3) how the abnormalities in hierarchical Bayesian representations are related to reduced flexibility. Figure 11 summarizes the results for these questions.

### 4.1. Reduced flexibility and pathology of ASD

The normal and weak (high stochasticity) meta-prior conditions did not show reduced flexibility regardless of external environment condition. In contrast, the networks with strong meta-prior (low stochasticity) condition showed less behavioral and cognitive flexibility in the stable environment. On the other hand, the noisy environment improved the behavioral flexibility under strong meta-prior condition.

This result of reduced flexibility under strong meta-prior condition is consistent with the finding reported by Wirkuttis and Tani (59) that the PV-RNN with higher meta-prior had stronger intention and less flexible interaction with others because the top-down prior belief had more effects on generated behaviors than bottom-up sensory signals. In addition to reproducing this finding, we found that behavioral flexibility was improved by increasing stimulus noise under the strong meta-prior condition. From an information theory view of PV-RNN, the network with strong meta-prior condition underestimates the reconstruction errors and overestimates the regularization errors in the loss function compared to the other conditions. The reason why the flexibility improved under noisy environment was that increasing stimulus noise led to an increase of reconstruction errors, resulting in amelioration of the balance between the reconstruction and regularization errors. Therefore, the combination of appropriate meta-prior and noise levels in the environment seems to be important for the flexible behavior. An alternative explanation for this amelioration effect is that increasing stimulus noise worked similar to the machine learning techniques to improve generalization capability such as augmentation (79) or denoising (80).

The findings that low stochasticity dynamics was related to reduced flexibility may provide new insights into the hypothesis that neural noise is involved in the formation of ASD. Previous theoretical studies have proposed conflicting hypotheses: one is there is more noise in the brain of people with ASD (72) and another is low noise in the brain of people with ASD (71). Our results support the hypothesis that low neural noise is associated with ASD. Furthermore, these results are consistent with the experimental findings using magnetic resonance imaging and electroencephalography that lower neural noise was associated with worse task performance in a typical developmental group (81, 82) and that lower neural noise was observed in ASD (73, 74). Moreover, in the Supplementary Results 2.3, we reported that some networks with low stochasticity dynamics generated sequences similar to restrictive and repeated behaviors. However, some studies have reported high neural noise in ASD (6, 7). Indeed, much noise intuitively seemed to lead to unstable and chaotic predictions and reduced task performance. The reason why the network with lower stochastic dynamics did not show inflexibility is that the flexibility task demanded to predict only one-step-ahead. For this reason, even if the disturbance of network dynamics by neural noise occurred, the network could sufficiently modify the predictions using observations. If the networks were required to predict a more longer future than one step, the noise would accumulate in the neural network, and the performance of the task is likely to deteriorate (55). It remains unclear why the higher neural noise induced better task performance in typical development but more severe symptoms in ASD, and refining experimental settings may contribute to solve this question.

In addition, the amelioration effect of environmental noise for flexibility was a novel finding of the current study. Indeed, although the effects of environment in developmental learning in ASD has been clinically well known (16, 17), there are few studies directly testing this topic from the computational aspect. For example, some studies discussed the environmental effects on mental disorders using computational theories only at the conceptual level (30, 31, 83). Our study demonstrated empirically that if the networks possessed risks for reduced flexibility, such as low stochasticity in neural dynamics, they could be ameliorated by increasing ambiguity in the external environment. On the other hand, clinical findings suggest that structuring the environment and removing ambiguity in stimulus were effective for people with ASD (16, 17). Although these findings may seem contradictory, our findings do not necessarily conflict with clinical findings, because many exposure methods for anxiety disorders have suggested that increasing prediction errors was important for correcting mislearning (84). Given the hypothesis that ASDs have a higher aversion to prediction errors, it is possible that these interventions, such as structuring the environment, do not contribute to learning, but only to emotional stabilization.

### 4.2. Acquisition of hierarchical and probabilistic representation

The current study demonstrated that stochasticity of neural dynamics (controlled by the level of meta-prior) was indeed associated with acquisition of the internal representations reflecting hierarchical and probabilistic environment structures. The neural network model under normal meta-prior condition could acquire the hierarchical and probabilistic representations in terms of passive inference (cognitive flexibility) and active generation (generative hierarchy). However, under weak meta-prior condition, there was an anomaly in the active decoding process rather than in the passive encoding process; namely, cognitive flexibility showed good performance, although the generative hierarchy in the higher layer showed poor scores. This may be because the learning of the prior distribution (used in the LST) did not progress as well as the posterior distribution (used in the test phase). As the properties of PV-RNN, the posterior distribution learns more easily and quickly than the prior distribution because the posterior distribution can use adaptive variables *a*_*t*_ in addition to neural dynamics units *d*_*t*_. Furthermore, under weak meta-prior condition, excessive neural noise might interfere information transmission to the higher layer from the lower layer and inhibit learning in the higher layer.

These results of simulation experiments can provide several insights for understanding the altered uncertainty estimation process assumed in ASD (29–32). The current experiment demonstrated that the mean unit in PV-RNN encoded higher-order probability (transition bias) in data sequences, and the variance units in the lower-order layer encoded sensorimotor noises (signal noise). This is not perfectly consistent with the predictive coding theory suggesting that the human brain represents uncertainty in the environment using the precision (inverse of variance) of Gaussian distribution (24). This inconsistency may be simply because of the fact that the higher-order hidden variables in the environment followed Bernoulli distribution and therefore neural networks did not need to use the variance units. However, there is still a possibility that the role of precision, as indicated by predictive coding theory, may be too normative. In fact, in a hierarchical neural network, estimation of precision can have broader effects beyond the weighting of information values assumed in the conceptual level of predictive coding theory such as disturbing neural dynamics observed in the weak meta-prior condition. Investigations using neural network implementation of predictive coding theory can contribute to further understanding of the roles of precision estimation and its alternation in developmental disorders.

The hierarchical Bayesian model has been treated as a very general and rational cognitive model for performing numerous tasks (26, 27). However, a hierarchical Bayesian model has been constructed by researchers a priori, and acquisition of representations reflecting the hierarchical Bayesian model have not been sufficiently addressed in cognitive neuroscience (33–35). In the area of machine learning and neurorobotics, although some studies focused on acquisition of hierarchical or probabilistic representations, these have some limitations. For example, some studies focusing on hierarchical representations did not assume sequential data because of using a variational auto-encoder (78, 85, 86) and did not use stochastic dynamics in RNN (42). Although there is research investigating internal representation using PV-RNN, the previous studies used lower-order probability (e.g., target state and signal noise) and did not consider explicitly higher-order probabilistic variables such as transition bias (55–60). The current result showing that artificial neural network models can acquire hierarchical Bayesian representations in a self-organizing manner is a crucial step to understanding underlying mechanisms for embedding the hierarchical Bayesian model into the brain system through developmental learning. Furthermore, our proposed research framework has applicability to a wide range of behavioral and cognitive phenotypes if its latent cognitive processes can be described using the Bayesian method (25, 26), for example, signal detection theory and drift-diffusion model in decision-making tasks.

### 4.3. Relationships between multiple developmental factors

It was observed that changes in the acquisition of hierarchical Bayesian representation did not necessarily induce inflexibility. Indeed, additional analysis demonstrated that the positive association between hierarchical representations (generative hierarchy) and behavioral flexibility was found only under strong meta-prior condition (Supplementary Results 2.1). However, under weak meta-prior condition, the behavioral and cognitive flexibility was comparable to normal meta-prior condition, but generative hierarchy in the higher layer was significantly lower.

This coexistence of good task performance and poor representation in the weak meta-prior condition is remarkable because the observable phenomena in performing tasks was equivalent while the underlying mechanism behind performing tasks was different between normal and weak meta-prior conditions. This finding is conceptualized as the issue of “equifinality” and “multifinality,” which are fundamental difficulties in understanding neurodevelopmental disorders (87). In particular, multiple factors leading to one developmental disorder exist (equifinality, for example, genetically distinct individuals may develop common social dysfunction), and conversely, the same cause may result in diverse and heterogeneous phenotypes (multifinality, for example, a particular gene can be associated with distinct psychiatric disorders).

From the aspect of equifinality, possible pathways other than the manipulations of meta-prior and signal noise leading to inflexibility were investigated (Supplementary Results 2.2–2.4). Specifically, the effects of different learning lengths were tested, motivated by the theoretical hypothesis that autistic characteristics in perception and cognition can be understood as “over-learning/over-fitting” (88). This additional experiment showed that the excessive learning length led to reductions in behavioral and cognitive flexibility (Supplementary Results 2.2, 2.3). Furthermore, from the aspect of episodic psychiatric disorders, even after normal development of hierarchical representation, altered flexibility can occur. To simulate this situation, we confirmed that hyper- and hypo-prior distributions (89) in the test phase can also induce inflexibility (Supplementary Results 2.4). Therefore, the reduced flexibility was caused both by alterations in the long-term developmental learning process (alterations of meta-prior, signal noise, and the learning length) and by abnormal prior influences in the short-term test phase. These simulations may contribute to constructing a unified explanation of inflexibility, which is a transdiagnostic phenotype observed in not only developmental disorders but also episodic mental disorders such as depression and schizophrenia.

It is also important that the simulations under strong meta-prior condition suggested that our proposed method can provide computational simulation frameworks for investigating multifinal phenomenon including treatment effects. Namely, the differences in external environmental stimulus induced the differences in generative hierarchy and flexibility under strong meta-prior condition, although settings of the individual network between environmental conditions were the same.

Equifinality and multifinality are widespread not only in developmental disorders but also in mental disorders and threaten the validity of the current diagnosis classification system (90). Resolving this problem may lead to the development of an effective intervention strategy that considers the individual differences (precision psychiatry), and the research handling equifinal and multifinal nature has been desirable. We are convinced that the proposed research framework contributes to understanding the multiple pathways leading to mental disorders.

### 4.4. Limitation and future directions

The simulation experiments had some limitations, which should be investigated in future research. First, the proposed framework is limited to ‘*in silico* simulation, and the findings obtained in the proposed framework are exploratory hypotheses. Therefore, the findings ‘*in silico* simulation should be verified with real data. For example, findings in the current experiments suggest that flexibility and/or hierarchical representation are impaired under strong and weak meta-prior conditions, suggesting that ASD may be a heterogeneous disorder. Given that flexibility was significantly reduced and hierarchical representation learning was impaired under the strong meta-prior condition, the neural dynamics with severe ASD may be low stochastic (highly deterministic). Conversely, mild ASD individuals, whose performance in flexibility task are close to the typical development group and who do not explicitly exhibit restrictive and repeated behaviors, may have high stochasticity in neural dynamics and may have problems with top-down predictions. These exploratory hypotheses could be verified using real data to refine the proposed framework.

The proposed framework has the potential to be extended for more diverse experimental settings beyond the simulations conducted in this study. For example, as mentioned above, cognitive-behavioral tasks other than the flexibility task are also applicable to the proposed framework. Furthermore, the direct effects of altered biological features other than meta-prior must be investigated in our framework as prior works on ASD and schizophrenia using neural network model utilized various virtual lesion to neural system (51–54). Moreover, the sequential data also has room for improvement. In the current study, the sequential data was two-dimensional and insufficient to reflect the real environment and sensorimotor signals. To overcome this problem, using neurorobotics experiments in which humanoid robots are used to interact with the external world to collect sensorimotor (e.g., vision and proprioception) signals would be useful (51, 52, 54, 91). Although the simulation experiments were still simple and were not sufficient to describe the interactions between multiple factors, these extended experiments based on the proposed framework will contribute to a deeper understanding of complex developmental processes.

In the simulation experiments, there were several technical issues. For example, the meta-prior, which was manipulated in experiments, was used as the hyper-parameter, which controls the stochasticity in neural dynamics. The relationship between meta-prior and stochasticity was confirmed in prior research (55) and in our simulations (Supplementary Figures S1, S2). However, meta-prior affects neural dynamics through mediating loss function rather than directly. Therefore, the process that meta-prior affected neural dynamics was more complex, and the roles of manipulating meta-prior required more careful discussion.

It was also unclear how to decide the meta-prior in the test phase, which affected the strength of prior belief. These values were decided by experimenter's trial and error in our study. The experimental results suggest that appropriate prior strength is required for good performance in both behavioral and cognitive flexibility (Supplementary Results 2.4); This is probably because it is better to ignore the prior information and use a copy of the last observations to enhance only behavioral flexibility. On the other hand, when inferring latent states, such as cognitive flexibility, both higher-order prior knowledge and observation are important to avoid adapting to accidental changes rather than true context switching. Therefore, there was a trade-off between behavioral and cognitive flexibility, and the system controlling exact prior strength may exist in humans and animals. Mathematically, this calculation may be automatically executed using Bayesian optimization or the prediction errors in the previous time step, such as deep active inference (39).

Furthermore, the variances of metrics, particularly cognitive flexibility and generative hierarchy, were big even in the same condition. The unstable results of learning were reported in the deep learning domain and often observed in the representation learning (92). Reducing these high variances is a new and important topic that needs to be discussed in both artificial neural networks and cognitive neuroscience domains.

### 4.5. Conclusion

In this study, to understand the relationships among hierarchical Bayesian representation, neural dynamics, the environment, and behavioral phenotype in developmental disorders, we proposed a new framework combining PV-RNN and the environment with hierarchical generative process. Through the experiments using this framework, we investigated whether inflexibility resulted from various factors (e.g., stochasticity in neural dynamics and the level of noises included in the environmental stimulus) with focus on hierarchical Bayesian representation learning. As a result, we found that the networks with normal stochastic dynamics acquired hierarchical and probabilistic representation reflecting the environmental structures and adapted flexibly to the new environment. Furthermore, we found that even if the networks possessed risks for reduced flexibility, such as low stochasticity in neural dynamics, they could be ameliorated by increasing ambiguity in the external environment. The networks with high stochastic dynamics had the hierarchical representations in terms of passive inference but did not have sufficient hierarchical and disentangled representations in terms of active generation. Therefore, our proposed method is useful for understanding atypical development such as reduced flexibility observed in ASD by bridging multiple factors including the neural dynamics, acquisitions of hierarchical representation, and the external environment.

## Data availability statement

The data presented in the study are deposited in the Github repository, which could be accessed via https://github.com/ncnp-cpsy/SimulatingDevelopmentalDiversity.git.

## Author contributions

TS, JT, and YY designed the experiment and analysis. TS performed the experiment and analyzed the data. AA developed the model and advised on the programming of the experiment. TS, AA, JT, MH, TH, and YY wrote the manuscript. All authors contributed to the article and approved the submitted version.
