# Supplementary material for: Simulating developmental diversity: Impact of neural stochasticity on atypical flexibility and hierarchy
Source: Front Psychiatry. 2023 Mar 15;14:1080668. doi: 10.3389/fpsyt.2023.1080668 (PMC10050443; doi:10.3389/fpsyt.2023.1080668)
Supplement: Supplementary file 1 [file Data_Sheet_1.PDF]

Supplementary Material

1 SUPPLEMENTARY METHOD

1.1 Details of Environmental Stimuli and Task Setting

The “target states” (i.e., LEFT or RIGHT) were sampled from the Bernoulli distribution parameterized by “transition bias.” The target coordinate points (i.e., the points of x- and y-axis) were sampled from multivariate Gaussian distributions whose mean parameters were set for each state (i.e., (-0.4, 0.35) in LEFT state, (0.4, 0.35) in RIGHT state) and whose covariance parameter reflected the “signal noise.” This covariance was a diagonal matrix, and its diagonal elements were set to 0.005 and 0.04 in stable and noisy environment condition, respectively. When returning to the HOME state from the target (LEFT or RIGHT) states, the target point was sampled from a Gaussian distribution with a mean of (0.0, -0.75) and a diagonal of covariance of 0.001.

The observable signal passed through seven points when the object moved from the current coordinate point to the target coordinate point. These seven points were sampled from the line connecting the current and target coordinate points following a uniform distribution, and a small amount of noise was added in the direction of the normal vector.

In the experiments, 18 sequences with 512 steps were used for training and testing. Two sets of nine sequences (18 sequences in total) were generated by nine different transition biases: 0.98, 0.10, 0.87, 0.21, 0.76, 0.32, 0.54, 0.43, and 0.65. Transition bias asymmetry was used to improve the divergence of variances in the sequences. If symmetric combinations of transition biases, such as 0.9 and 0.1, were used, the variances of sequences generated by these parameters were equal (i.e., 0.09). In the test sequences, the transition bias was randomly sampled after switching so that the transition bias in last-half period was always switching in the opposite directions of the transition bias in first-half period.

Switching of noise levels in flexibility tasks occurred when examining the interaction effects between meta-prior and signal noise and did not occur when comparing only effects of meta-prior. Specifically, test sequences were stable all times when only the networks learned in the stable environment condition were compared, while the noise level in test sequences switched at the middle point using both stable and noisy conditions.

1.2 Quantitative Metrics

In Table S1, generation strategies used for the quantification of each metric were summarized.

1.2.1 Behavioral and Cognitive Flexibility

To assess ASD (autism spectrum disorder) phenotype, we used two types of performance in flexibility task. One is how accurately network predicted observation signals (behavioral flexibility), and the other is how accurately network inferred unobservable hidden contexts (cognitive flexibility). Behavioral flexibility was the percentage of the agreements between the states of target signals and of predictions. Coordinates of sequences were converted to states as follows: $y \leq 0$ represents the HOME state, $y > 0$ and $x < 0$ represent the LEFT state, and $y > 0$ and $x \geq 0$ represent the RIGHT state. More common evaluation methods, such as mean square error, were not used, because they evaluated inappropriate predictions as overly good predictions (e.g., moving up and down along y-axis while staying at the center of the x-axis).

Cognitive flexibility is the correlation coefficient between true highest-order hidden variables (transition bias) in target sequences and the neural activities of latent mean units in the highest layer. In computing these correlations, each sequence in test (18 sequences in total) was merged along the time axis. The reason that the average of the 18 sequences was not used was to improve the accuracy of correlation estimation by increasing the variety of true hidden context (transition bias).

In the analysis and figures of test phase, the one-step-ahead mean parameters inferred by the prior model were plotted (namely, prediction, not postdiction, was analyzed).

1.2.2 Generative Hierarchy

We used the generations by latent space traversal when quantifying the goodness of representations in terms of active generation ability (generative hierarchy). We quantified the properties (e.g., the number of transitions to LEFT) of generated sequences when activities of one latent unit were fixed at a particular value. Furthermore, using correlation coefficients between the fixed activities and output properties, we made the similarity matrix between the latent units and the output properties. Next, for each latent unit, coding efficacy was defined using the maximum similarity. Then, the average of the coding efficacy of all latent units is considered to be the goodness of generative hierarchy in the network. When quantifying generative hierarchy in each layer, only the latent units in the layer were used. As the output properties, the number of transitions to each state, the number of stay steps in each state, and the variance in each state were used.

1.2.3 Reconstruction Ability

The similarities were used to evaluate how the generated sequences using prior generation reflected the observable probabilistic properties in training data. To evaluate the reconstruction ability of probabilistic property, the Kullbuck-Leibular divergence (KLD) between sequences used in learning phase and sequences generated by the networks was used. It is noted that the lower KLD reflects more similarity. We considered four types of reconstruction ability between training and generation sequences: plane, period, transition, and expanse similarity. For the analysis, the final 512 steps of 18 sequences with 10,000 steps generated by prior generation were utilized.

In the plane, period, and transition similarities, the experience frequency distributions (histogram) were calculated in each sequence. In the plane similarity, we divided the xy-plane into a 20 x 20 lattice and calculated the frequency distribution by counting the number of time steps spent in each grid. In the period similarity, we calculated the frequencies of half-periods of the y-axis movements in each sequence. The time point when the sign of $(y_t - y_{t-1})(y_{t-1} - y_{t-2})$ was negative was used for computing the half-period. In the transition similarity, the frequencies of nine transition patterns from the other to one state (HOME, LEFT, and RIGHT) were counted.

Furthermore, we developed expanse similarity to investigate whether the network could generate various predictions. In the expanse similarity, first, the ratios of the transition from HOME to LEFT in each of 18 sequences were calculated. Next, these ratios were translated into a histogram in which the interval from zero to one was divided into eight bins. The KLD between this histogram and uniform distributions was used as expanse similarity.

1.2.4 Neural Noise

We quantified the stochasticity of neural dynamics in the lower layer. The training sequences consisted of 16 time steps in one period, and it is expected that the period of neural activities was also around 16 time

steps. Therefore, when the mean activities of latent units in the lower layer during prior generations were fitted to the moving average model using the *seasonal_decompose* function of the *statsmodels* package in Python. Then, the sum of the residual terms was used as neural noise (Figure S1). The neural noise under each condition was shown in Figure S2.

1.3 Architecture and Generative Process of PV-RNN

1.3.1 Architecture and Initialization of PV-RNN

As explained in the main text, the PV-RNN has four probabilistic models: prior model $p(z_t|d_{t-1})$, posterior model $q(z_t|d_{t-1}, a_t)$, recurrent model $p(d_t|d_{t-1}, z_t)$, and generative model $p(x_t|d_t)$.

The PV-RNN $p(x_{\leq T}) = p(x_1, x_2, \ldots, x_T)$ was factorized as follows:

$$
\begin{aligned}
p(x_{\leq T}) &= \int \cdots \int p(x_{\leq T}, d_{\leq T}, z_{\leq T}) dd_{\leq T} dz_{\leq T} \\
&= \int \cdots \int p(x_1|d_1)p(d_1)p(z_1) \prod_{t=2}^{T} p(x_t|d_t)p(d_t|d_{t-1}, z_t)p(z_t|d_{t-1}) dd_{\leq T} dz_{\leq T}
\end{aligned}
$$

In addition, using the hierarchy of PV-RNN, these probabilistic models were further factorized as follows:

$$
\begin{aligned}
p(x_t|d_t) &= p(x_t|d_t^1) \\
p(d_t|d_{t-1}, z_t) &= p(d_t^1|d_{t-1}^1, d_{t-1}^2, z_t^1)p(d_t^L|d_{t-1}^{L-1}, d_{t-1}^L, z_t^L) \prod_{l=2}^{L-1} p(d_t^l|d_{t-1}^{l-1}, d_{t-1}^l, d_{t-1}^{l+1}, z_t^l) \\
p(z_t|d_{t-1}) &= \prod_{l=1}^{L} p(z_t^l|d_{t-1}^l) \\
q(z_t|d_{t-1}, a_t) &= \prod_{l=1}^{L} p(z_t^l|d_{t-1}^l, a_t^l)
\end{aligned}
$$

Then, data distribution of PV-RNN $p(x_{\leq T})$ was rewritten as follows:

$$
\begin{aligned}
p(x_{\leq T}) &= \int \cdots \int p(x_1|d_1^1) \prod_{l=1}^{L} p(d_1^l) \prod_{l=1}^{L} p(z_1^l) \\
&\prod_{t=2}^{T} \Big\{ p(x_t|d_t^1) \\
&\{ p(d_t^1|d_{t-1}^1, d_{t-1}^2, z_t^1) p(d_t^L|d_{t-1}^{L-1}, d_{t-1}^L, z_t^L) \prod_{l=2}^{L-1} p(d_t^l|d_{t-1}^{l-1}, d_{t-1}^l, d_{t-1}^{l+1}, z_t^l) \} \\
&\{ \prod_{l=1}^{L} p(z_t^l|d_{t-1}^l) \} \Big\} dd_{\leq T} dz_{\leq T}
\end{aligned}
$$

A unit Gaussian distribution was used instead of the prior model in the initial step $p(z_1)$, suggested by previous studies (1, 2). The initial values of hidden (neural dynamic) units d_1 (corresponding to sampling from $p(d_1)$) were obtained by computing a one-step forward pass calculation using $p(d_1|d_0, z_1)$ with $d_0 = 0$. As deterministic variables were used as d_t in PV-RNN, the integral of d_t was omitted in the following.

1.3.2 Reparameterization of PV-RNN as Neural Network Model

1.3.2.1 Recurrent Model

In PV-RNN (3), the hierarchical structure and function were archived using two sources. One is differing the time window of neural dynamics for each layer in the recurrent model $p(d_t|d_{t-1}, z_t)$ which conveys information from past to now.

$$
\begin{aligned}
h_t &= \frac{1}{\tau}(W_r d_{t-1} + W_{latent} z_t + b_r) + (1 - \frac{1}{\tau}) h_{t-1} \\
d_t &= \tanh(h_t)
\end{aligned}
$$

W_r represents the synaptic weight matrix between hidden units, which plays an important role in passing on information from the past to the present between layers. W_{latent} represents the matrix which codes the synaptic weights connecting to hidden units d_t from latent units z_t. b_r is the bias parameter vector of recurrent model. τ represents the time constant vector and determines the time window of information processing. Grouping time constants, for example, $\tau = (\tau^1, \tau^2, \tau^3)$ in three layers, contributes to a recurrent neural network model for self-organizing the functional hierarchy (4, 5). This is because of the fact that the speed of temporal dynamics varies depending on functional hierarchy and abstract level, for example, higher-order functions and more abstract constructs tend to change more slowly over time than concrete sensorimotor stimuli. In our experiments, τ was set to 2, 8, and 32 for units in the lower, middle, and higher layers, respectively.

In addition, constrained information flow by the structural disconnectivity contributes to the hierarchical feature of PV-RNN. In the recurrent model, hidden units in one layer d_t^l have connections to hidden units d_{t-1}^l and latent units z_t^l in the same layer, one level higher hidden units d_{t-1}^{l+1}, and one level lower hidden

units d_{t-1}^{l-1}, and other connections are disconnected (synaptic weights are set to zero). In the case of three layers as used in our study, connections between d_t units in the higher layer and in lower layer were turned off, for example, d_t in the higher layer was affected by only d_{t-1} in the higher and middle layers. On the other hand, d_t in the middle layer was determined using d_{t-1} in all layers. Therefore, the middle layer, which connects both of the top and bottom layer, plays a key role as a bottleneck in the hierarchical information flow. Other probabilistic models in PV-RNN also have these constraints of information flow as discussed in the following sections.

1.3.2.2 Prior and Posterior Model

Prior model $p(z_t|d_{t-1})$ is reparameterized as follows:

$$\begin{aligned} p(z_t|d_{t-1}) &= Norm(\mu_t^p, \sigma_t^{2^p}) \\ \mu_t^p &= \tanh(W_{p,\mu}d_{t-1} + b_{p,\mu}) \\ \sigma_t^{2^p} &= \exp(W_{p,\sigma}d_{t-1} + b_{p,\sigma}) \end{aligned}$$

$W_{p,\mu}$ and $W_{p,\sigma}$ are the synaptic weight matrix from d_{t-1} to the mean μ_t^p and variance $\sigma_t^{2^p}$ units of prior model, respectively. $b_{p,\mu}$ and $b_{p,\sigma}$ are bias parameter vectors for estimation of the mean and variance units of prior model, respectively.

Posterior model $q(z_t|d_{t-1}, a_t)$ is reparameterized as follows:

$$\begin{aligned} q(z_t|d_{t-1}, a_t) &= Norm(\mu_t^q, \sigma_t^{2^q}) \\ \mu_t^q &= \tanh(W_{q,\mu}d_{t-1} + a_{t,\mu} + b_{q,\mu}) \\ \sigma_t^{2^q} &= \exp(W_{q,\sigma}d_{t-1} + a_{t,\sigma} + b_{q,\sigma}) \end{aligned}$$

$W_{q,\mu}$ and $W_{q,\sigma}$ are the synaptic weight matrix from d_{t-1} to the mean μ_t^q and variance $\sigma_t^{2^q}$ units of posterior model, respectively. $b_{q,\mu}$ and $b_{q,\sigma}$ are bias parameter vectors for estimation of the mean and variance units of posterior model, respectively. a_t is used as the learnable parameters for each training sequence and each time step.

z_t are sampled as follows:

$$z_t = \mu_t + \sigma_t^2 * \epsilon$$

Here, ϵ is sampled from a unit Gaussian distribution only once per one inference. Whether prior or posterior model is used as μ_t and σ_t^2 depends on generation strategies (e.g., error regression and prior generation). In the figures in this study, the μ_t of the prior model was basically presented.

In addition, prior $p(z_t|d_{t-1})$ and posterior $q(z_t|d_{t-1}, a_t)$ models have disconnections similar to recurrent model. In these models, the latent states for each layer z_t^l were inferred using only the hidden units in the same layer d_{t-1}^l (e.g., z_t units in the higher layer inferred only using d_{t-1} unit in the higher layer).

1.3.2.3 Generative Model

The predictions x_t are generated using only hidden units in the lowest layer d_t^1. About observation signals, each dimension in original sequences (two-dimension) was converted to sparse 10-dimension data using softmax transformation (3). Thus, the generative model follows categorical distribution with 10 classes for each original dimension.

$$
\begin{aligned}
p(x_t|d_t) &= Cat(\lambda_t) \\
\lambda_t &= softmax(W_g d_t^1 + b_g)
\end{aligned}
$$

W_g represents the synaptic weight matrix from d_t, and b_g represents the bias parameter vector.

1.3.3 Generative and Inference Process

In our experiments, several types of generation strategies of the PV-RNN were used, such as error regression (used in the test phase), prior generation (called free generation sometimes), latent space traversal, and target generation. As mentioned before, a PV-RNN was constructed from four types of probabilistic models: prior model $p(z_t|d_{t-1})$, approximate posterior model $q(z_t|d_{t-1}, a_t)$, recurrent model $p(d_t|d_{t-1}, z_t)$, and generative model $p(x_t|d_t)$. Various generation strategies were implemented by changing the combination of these probabilistic models.

Both the prior and posterior models were used in the generation process including loss calculation (i.e., learning and error regression). The generation process in the learning process is as follows: first, the prior model $p(z_t|d_{t-1})$ inferred the mean and variance values of the latent variables z_t using only the hidden units d_{t-1} as the input, while the posterior model $q(z_t|d_{t-1}, a_t)$ inferred these values using a_t in addition to d_{t-1}. Then, the KLD between the prior and posterior distributions (regularization errors) was computed. After z_t was sampled from the posterior means and variances, d_t units were updated using the recurrent model $p(d_t|d_{t-1}, z_t)$. Next, using the generative model $p(x_t|d_t)$, predictions x_t were made, and the reconstruction errors between the predictions and observations were quantified.

Error regression used in the test phase is a sequential estimation (online inference) scheme based on the prediction error minimization process. This was the same as the learning process in that both the prior and posterior were used for calculation of the loss. However, for predictions of unknown future inputs, the latent variables z_t were inferred using only the prior model (similar to mental simulation because observations were not used). Subsequently, when the network received new inputs, then the loss was calculated, and the adaptive variables a_t of the posterior were modified by fixing synaptic weights using error backpropagation. By repeating this process, the latent variables z_t in posterior were corrected to appropriate values (postdiction). These updated latent variables were used to predict future data using prior again. In the experiments, the loss was calculated using the period ranging from 15 time steps in the past to one time step in the future. The number of iterations required for updating was set to 20.

Other generation strategies were used for analysis of hierarchical representation and reconstruction abilities. In prior generation, external inputs and prediction errors were not used, and generations were based on the closed-loop of neural dynamics. Particularly, the latent states at the current time z_t were inferred using only the prior model $p(z_t|d_{t-1})$ where only hidden units at the previous time d_{t-1} determined the latent states, and then the hidden units d_t and observations x_t were generated using

the recurrent model $p(d_t|d_{t-1}, z_t)$ and generative model $p(x_t|d_t)$. Prior generation was used to examine whether the network could reconstruct the characteristics of the learned sequences.

Latent space traversal is similar in that it does not use a posterior model; however, the value of one target latent unit was fixed and changed intentionally by the researchers, instead of using the prior model (similar to manipulation for single neuron). In this way, the information decoded by the target neuron was investigated in the latent space traversal. For each target neural unit, sequences of 1,024-time steps were generated by setting the activity of the target neural unit at a particular fixing value (21 values in the interval from -1.0 to 1.0). Figure S3 depicted examples of latent space traversal under the normal meta-prior condition in which decreasing the activity of unit4 increased transitions to the LEFT state and increasing the activity of unit4 increased transitions to the RIGHT state.

In target generation, to infer the latent states, the posterior model $q(z_t|d_{t-1}, a_t)$ was used only in the initial step; the prior model $p(z_t|d_t)$ was used in other steps. The adaptive variables a_t obtained in the learning process were used in target generation, while a_t were sequentially inferred in error regression. The training sequences corresponding to a_t were reconstructed using target generation when learning progressed sufficiently. This is because the neural network acquired initial value sensitivity in which the time evolution of the neural dynamics was determined only in the initial values. The target generation was used to examine the over-fitting of each network.

1.4 Deviation of Loss Function in PV-RNN

Generally, statistical model $p_{model}(x)$ learns its parameters to approximate the true data distribution $p_{data}(x)$. When using KLD, the similarity between these two distributions is represented as follows:

$$
\begin{aligned}
D_{KL}[p_{data}(x)||p_{model}(x)] &= \int p_{data}(x) \log \frac{p_{data}(x)}{p_{model}(x)} dx \\
&= \int p_{data}(x) \log p_{data}(x) dx \\
&\quad - \int p_{data}(x) \log p_{model}(x) dx \\
&= -H[p_{data}(x)] - E_{p_{data}(x)}[\log p_{model}(x)]
\end{aligned}
$$

In this equation, the first term represents entropy in true data distribution $p_{data}(x)$ and is an unknowable constant value. By replacing the expectation in the second term into empirical distribution, this equation suggests that maximizing $\log p_{model}(x)$, which is called log marginal likelihood and evidence, leads to minimizing the similarity as an average.

From the maximization of evidence, the evidence lower bound (ELBO) can be derived, and the loss function of PV-RNN is negative of ELBO (similar to variational autoencoder (6, 7)). To simplify the notation while keeping the generalizability to PV-RNN and other variational RNNs (3, 8), we consider a probabilistic model as in Figure S4, where z_t and x_t represent latent and observed variables, respectively. In this case, the evidence to be maximized is a $\log p(x_{\leq T}) = \log p(x_1, x_2, \ldots, x_T)$.

$$
\begin{aligned}
\log p(x_{\leq T}) &= \log \int \cdots \int p(x_{\leq T}, z_{\leq T}) dz_{\leq T} \\
&= \log \int \cdots \int q(z_1|x_1) \frac{p(x_{\leq T}, z_{\leq T})}{q(z_1|x_1)} dz_{\leq T} \\
&\geq \int q(z_1|x_1) \log \int \cdots \int \frac{p(x_{\leq T}, z_{\leq T})}{q(z_1|x_1)} dz_{\leq T} \\
&= \int q(z_1|x_1) \log \int \cdots \int q(z_2|x_{\leq 2}, z_{<2}) \frac{p(x_{\leq T}, z_{\leq T})}{q(z_1|x_1) q(z_2|x_{\leq 2}, z_{<2})} dz_{\leq T} \\
&\geq \int \int q(z_1|x_1) q(z_2|x_{\leq 2}, z_{<2}) \log \int \cdots \int \frac{p(x_{\leq T}, z_{\leq T})}{q(z_1|x_1) q(z_2|x_{\leq 2}, z_{<2})} dz_{\leq T} \\
&\vdots \\
&\geq \int \cdots \int q(z_1|x_1) \prod_{t=2}^{T} q(z_t|x_{\leq t}, z_{<t}) \log \frac{p(x_{\leq T}, z_{\leq T})}{q(z_1|x_1) \prod_{t=2}^{T} q(z_t|x_{\leq t}, z_{<t})} dz_{\leq T} \\
&= \int \cdots \int q(z_{\leq T}|x_{\leq T}) \log \frac{p(x_{\leq T}, z_{\leq T})}{q(z_{\leq T}|x_{\leq T})} dz_{\leq T} \\
&= E_{q(z_{\leq T}|x_{\leq T})} [\log \frac{p(x_{\leq T}, z_{\leq T})}{q(z_{\leq T}|x_{\leq T})}] \\
&= ELBO
\end{aligned}
$$

In this derivation, the posterior model $q(z_1|x_1), q(z_2|x_{\leq 2}, z_{<2}), \ldots, q(z_T|x_{\leq T}, z_{<T})$ are introduced to approximate true posterior $p(z_1|x_1), p(z_2|x_{\leq 2}, z_{<2}), \ldots, p(z_T|x_{\leq T}, z_{<T})$, respectively and repeatedly. Furthermore, we used Jensen's inequality $f(E[x]) \geq E[f(x)]$ for a concave function, repeatedly. It has been shown that if the equality of this inequality holds, the true and approximate posterior distribution will coincide.

Furthermore, noting that the distribution of the complete data is:

$$
\begin{aligned}
p(x_{\leq T}, z_{\leq T}) &= p(x_1|z_1) p(z_1) \prod_{t=2}^{T} p(x_t|x_{<t}, z_{\leq t}) p(z_t|z_{<t}) \\
&= \{p(x_1|z_1) \prod_{t=2}^{T} p(x_t|x_{<t}, z_{\leq t})\} \{p(z_1) \prod_{t=2}^{T} p(z_t|z_{<t})\} \\
&= p(x_{\leq T}|z_{\leq T}) p(z_{\leq T}).
\end{aligned}
$$

Then, ELBO can be expanded as follows:

$$
\begin{aligned}
ELBO &= E_{q(z_{\leq T}|x_{\leq T})}[\log \frac{p(x_{\leq T}, z_{\leq T})}{q(z_{\leq T}|x_{\leq T})}] \\
&= E_{q(z_{\leq T}|x_{\leq T})}[\log \frac{p(x_{\leq T}|z_{\leq T})p(z_{\leq T})}{q(z_{\leq T}|x_{\leq T})}] \\
&= E_{q(z_{\leq T}|x_{\leq T})}[\log p(x_{\leq T}|z_{\leq T}) - \log \frac{q(z_{\leq T}|x_{\leq T})}{p(z_{\leq T})}] \\
&= E_{q(z_{\leq T}|x_{\leq T})}[\log p(x_{\leq T}|z_{\leq T})] - E_{q(z_{\leq T}|x_{\leq T})}[\log \frac{q(z_{\leq T}|x_{\leq T})}{p(z_{\leq T})}] \\
&= E_{q(z_{\leq T}|x_{\leq T})}[\log p(x_{\leq T}|z_{\leq T})] - D_{KL}[q(z_{\leq T}|x_{\leq T})||p(z_{\leq T})]
\end{aligned}
$$

Next, we try to decompose joint distribution into distributions of each time step to infer sequentially as follows:

$$
\begin{aligned}
ELBO &= E_{q(z_{\leq T}|x_{\leq T})}[\log p(x_{\leq T}|z_{\leq T})] - D_{KL}[q(z_{\leq T}|x_{\leq T})||p(z_{\leq T})] \\
&= E_{q(z_{\leq T}|x_{\leq T})}[\log p(x_1|z_1)\prod_{t=2}^{T} p(x_t|x_{<t}, z_{\leq t})] \\
&\quad -E_{q(z_{\leq T}|x_{\leq T})}[\log \frac{q(z_1|x_1)\prod_{t=2}^{T} q(z_t|x_{\leq t}, z_{<t})}{p(z_1)\prod_{t=2}^{T} p(z_t|z_{<t})}] \\
&= E_{q(z_{\leq T}|x_{\leq T})}[\log p(x_1|z_1) + \sum_{t=2}^{T} \log p(x_t|x_{<t}, z_{\leq t})] \\
&\quad -E_{q(z_{\leq T}|x_{\leq T})}[\log \frac{q(z_1|x_1)}{p(z_1)} + \sum_{t=2}^{T} \log \frac{q(z_t|x_{\leq t}, z_{<t})}{p(z_t|z_{<t})}] \\
&= E_{q(z_1|x_1)}[\log p(x_1|z_1)] + \sum_{t=2}^{T} E_{q(z_{\leq t}|x_{\leq t})}[\log p(x_t|x_{<t}, z_{\leq t})] \\
&\quad -E_{q(z_1|x_1)}[\log \frac{q(z_1|x_1)}{p(z_1)}] - \sum_{t=2}^{T} E_{q(z_{\leq t}|x_{\leq t})}[\log \frac{q(z_t|x_{\leq t}, z_{<t})}{p(z_t|z_{<t})}] \\
&= E_{q(z_1|x_1)}[\log p(x_1|z_1)] + \sum_{t=2}^{T} E_{q(z_{\leq t}|x_{\leq t})}[\log p(x_t|x_{<t}, z_{\leq t})] \\
&\quad -E_{q(z_1|x_1)}[\log \frac{q(z_1|x_1)}{p(z_1)}] - \sum_{t=2}^{T} E_{q(z_{\leq t-1}|x_{\leq t-1})}[\int q(z_t|x_{\leq t}, z_{<t}) \log \frac{q(z_t|x_{\leq t}, z_{<t})}{p(z_t|z_{<t})} dz_t] \\
&= E_{q(z_1|x_1)}[\log p(x_1|z_1)] + \sum_{t=2}^{T} E_{q(z_{\leq t}|x_{\leq t})}[\log p(x_t|x_{<t}, z_{\leq t})] \\
&\quad -D_{KL}[q(z_1|x_1)||p(z_1)] - \sum_{t=2}^{T} E_{q(z_{\leq t-1}|x_{\leq t-1})}[D_{KL}[q(z_t|x_{\leq t}, z_{<t})||p(z_t|z_{<t})]]
\end{aligned}
$$

A negative version of ELBO is the loss function of PV-RNN (3) and other variational RNN (8). When converting this generalized model in Figure S4 to the PV-RNN, we must replace three probabilistic distributions, as shown in Table S2. Hidden units d_t in PV-RNN, which were included in the latent variables z_t in the generalized model, were basically omitted because they were deterministic variables and removed by the integral.

The latent variables z_t at time t were sampled only once and only for that time step to reduce computation cost. Accurately, the expectation using z_t of all time steps from 1 to t should be taken because $q(z_{\leq t}|x_{\leq t}) = q(z_1|x_1)\prod_{k=2}^{t} q(z_k|x_{\leq k}, z_{<k})$. The loss function in the main text can be derived by introducing the meta-prior w. It is noted that the loss function in the main text does not distinguish between the initial and subsequent time steps (from 2 to T).

In our experiment, all parameters of PV-RNN (the synaptic weights and adaptive variables a_t) were optimized using backpropagation through time. In this study, the meta-priors for three layers were set to 0.01, 0.01, and 0.01 at the first time step and 1.0, 1.0, and 1.0 at other time steps under the normal meta-prior condition. In the test phase (inference process using error regression), the meta-priors were set to 0.001, 0.01, and 0.1. As an optimizer, Adam (9) was used, and its learning rates, β_1 and β_2, were set to 0.001, 0.9, and 0.999 in learning and 0.09, 0.5, and 0.999 in the test phase, respectively.

2 SUPPLEMENTARY RESULTS

2.1 Representation Learning and Flexibility

The remaining question was how the hierarchical representation acquisition related to inflexibility. For this purpose, the correlation and moderation analysis were performed to clarify the relationships between meta-prior, noise level in observation signals, generative hierarchy, and behavioral flexibility.

The results of correlation analysis revealed the significant association between generative hierarchy and behavioral flexibility when all conditions were combined without distinguishing groups (r = 0.4744; p $<$ 0.0001). However, the scatter plot (Figure S5) suggests that the effect of meta-prior conditions in the learning process was greater than that of generative hierarchy, and the sign and strength of the associations between generative hierarchy and behavioral flexibility varied based on the learning condition (r = -0.1756; p = 0.5153 in normal, r = 0.7411; p = 0.0003 in strong, r = 0.0715; p = 0.7711 in weak meta-prior conditions under stable environment, r = 0.2970; p = 0.2169 in normal, r = 0.1178; p = 0.6639 in strong, r = 0.1228; p = 0.6062 in weak meta-prior conditions under noisy environment). For correlation analysis, the R package "psych" (10) was used.

Thus, we constructed a multiple regression model in which the developmental factors (i.e., meta-prior and noise level in observation signals) and these interactions moderated the effect of generative hierarchy in all layers on behavioral flexibility (Figure S6). For moderation analysis, the R package "stats" (11) was used. The results of the analysis (Table S3) showed that generative hierarchy had significant effects on behavioral flexibility under only strong meta-prior conditions (β = 0.8397, p = 0.0211), and the main effect of generative hierarchy was not significant (β = -0.2867, p = 0.4109). These results implicate the goodness of hierarchical representation quantified using generative hierarchy has positive effects on autistic phenotype under limited situations.

2.2 Excessive Learning Length Condition

In addition to manipulations of meta-prior and signal noise presented in main text, the length (number of iterations) of the developmental learning phase was manipulated. This was based on the previous theoretical hypothesis that autistic characteristics in perception and cognition can be understood as "over-learning/over-fitting" (12). The networks under the long learning condition were obtained by performing additional learning for the networks under the normal meta-prior condition. The length of the learning phase was 200,000 and 10,000 under the long learning and normal meta-prior condition, respectively. Analyses of variance (ANOVA) were used for comparing conditions (four levels, normal, strong, weak meta-prior, and long learning).

Figure S7 depicted the predictions and neural activities in the flexibility task under the long learning condition. Similar to the strong meta-prior condition, the failures to match movement timing and perseveration errors were observed under the long learning conditions. The quantitative evaluations supported these observations, as shown in Figure S8. Both behavioral (F(3, 70) = 89.9353; $p < 0.0001$ in

ANOVA) and cognitive (F=(3, 75) = 27.8443; $p < 0.0001$ in ANOVA) flexibility were reduced under the long learning condition vs. the normal meta-prior condition. Therefore, the reductions in flexibility were induced by a variety of factors, not limited to the strong meta-prior condition (equifinality).

However, Figure S9 shows that the results of generative hierarchy under the long learning condition was similar to that of the normal meta-prior condition ($F(3, 75) = 202.2893$; $p < 0.0001$ in lower layer, $F(3, 70) = 39.5485$; $p < 0.0001$ in middle layer, $F(3, 71) = 22.6098$; $p < 0.0001$ in higher layer, $F(3, 76) = 0.0658$; $p = 0.9779$ in noise representation). The significant superior relationships did not exist between the normal meta-prior and long learning conditions in the post hoc tests. Therefore, under the long learning condition, higher-order representations were good for active generation (latent space traversal) but bad for passive inference (error regression), in contrast to these of the weak meta-prior condition.

2.3 Restrictive and Repetitive Behavior and Reconstruction Ability

In addition to the results in the main text, some networks with the strong meta-prior condition did not modify the predictions based on minimization of prediction errors and repeated one transition outputs ignoring target sequences (Figure S10), similar to restrictive and repetitive behaviors observed in the patients with ASD. This observation indicated that the networks under the strong meta-prior condition did not acquired prior model which reflects probabilistic structures in the environment, and the mechanism leading to inflexibility under the strong meta-prior condition differed from long learning condition. Therefore, in this section, we analyzed whether the networks could generate predictions that reflected the probabilistic properties in observed signals using only prior distributions (i.e., the reconstruction ability using prior distributions). In this generation strategy, the networks inferred hidden states and predicted observations using only internal neural dynamics rather than external input and prediction errors.

Figure S11, S12, S13, S14, S15 present the predictions and latent units (average unit) generated under each condition using prior generation. As seen from the generated sequence and state transition in Figure S11, the networks under normal meta-prior acquired the transition pattern, including training data (e.g., HOME $\rightarrow$ LEFT), and never generated the transition patterns that did not exist in training data (e.g., RIGHT $\rightarrow$ LEFT). The periods in the generated sequence were similar to training sequences (approximately 16 time steps), although the variability in the 2-dimensional plane plot seemed to be slightly smaller than that of the training data. To investigate whether the network dynamics were pulled into the attractor, we made one network under normal meta-prior conditions to generate 18 sequences using different random seeds. The results are shown in Figure S16B, where each dot represents the LEFT transition ratio of one sequence. The ratios of the LEFT transition by the network were as diverse as the training data (left in Figure S16B), which suggests that the predictions by the network were not pulled into the attractor. Therefore, the prior generations by a normal meta-prior neural network were able to properly reflect the observable characteristics of the training data.

Some networks under the strong meta-prior condition repeatedly generated the middle points between the LEFT and RIGHT states sometimes (Figure S15), although other networks generated normally (Figure S12). This generation strategy, repeating middle points, was very rational in that it reduced mean squared errors because the target states following probabilistic rule could not be perfectly predicted. In addition, even if the initial values of each neuron were set to different values using different random seeds, the generations were the same (right in Figure S16B). Therefore, the prediction patterns observed in strong meta-prior conditions (Figure S10, S15) were sometimes similar to restricted and repeated behaviors observed in patients with ASD because this network persisted in generating only one pattern.

In contrast, the weak meta-prior condition was able to generate transition patterns and variability in the plane plot in the training data; however, it included considerable noise, and the period was unstable (Figure S13). Generations under long learning conditions were similar to those under normal meta-prior conditions (Figure S14).

Subsequently, we quantitatively confirmed these properties. To investigate the variances in the plane plot, the two-dimensional observations were converted to frequencies in 20 x 20 lattices, and the KLD between outputs and training data was calculated. Similarly, the frequencies of nine transition patterns (e.g., HOME $\rightarrow$ LEFT, RIGHT $\rightarrow$ LEFT) and periods were calculated, and the KLD between the outputs and training data was quantified for each metric. The results (Figure S16A) showed almost normal meta-prior conditions, and long learning conditions appropriately generated observations that reflected training sequences. Specifically, only under strong meta-prior conditions, the transition reconstruction was reduced ($F(3, 71) = 7.2567$; $p = 0.0003$ in ANOVA, $t(71) = 3.7418$; $p = 0.0014$ at normal $<$ strong, $t(71) = 3.6744$; $p = 0.0014$ at long learning $<$ strong, and $t(71) = 3.8731$; $p = 0.0014$ at weak $<$ strong using post-hoc test) and the reconstruction of frequencies in the two-dimensional plane ($F(3, 68)= 11.1106$; $p < 0.0001$ in ANOVA, $t(68) = 4.0230$; $p = 0.0004$ at normal $<$ strong, $t(68) = 4.0093$; $p = 0.0005$ at long learning $<$ strong, and $t(68) = 5.3680$; $p < 0.0001$ at weak $<$ strong using post-hoc test) was reduced. In period reconstruction, the normal and strong meta-prior conditions were better than the week meta-prior condition ($F(3, 69)= 7.4315$; $p = 0.0002$ in ANOVA, $t(69) = 4.3079$; $p = 0.0003$ at normal $<$ weak, $t(69) = 3.6786$; $p = 0.0014$ at strong $<$ weak using post-hoc tests). There were no significant differences in the number of generated sequences, which were biased to the LEFT ($F(3, 73) = 0.1435$; $p = 0.9335$), probably because variances within conditions were high.

Furthermore, Figure S16C shows the learning curve of the reconstruction ability, which was quantified using state agreements between target (i.e., training or test) sequences and the PV-RNN under normal meta-prior conditions. For the reconstruction ability of training sequences (left side in Figure S16C), target generation was used as the generation strategy. For the reconstruction ability of test sequences (generalization ability in Figure S16C), the test sequences that did not include context (transition bias) switching were used to equalize the properties of target sequences between training and test. The results suggests that networks with long learning conditions over-fit the training sequences. This network showed good performance in a known environment instead of the capacity to adapt to a variety of situations, including an unknown environment, a theory known as "bias-variance trade-off."

Reductions in behavioral and cognitive flexibility were observed in both long learning and strong meta-prior conditions, but restricted and repeated generation patterns were observed only under the strong meta-prior condition. The results indicated that restricted and repeated behaviors observed in ASD may be interpreted as abnormal acquisitions of prior model reflecting probabilistic structure in the environment. In contrast, the long learning condition was good at reconstructing training sequences, but its generalization abilities were low. This suggests that the mechanism of reduced flexibility involves multiple paths (equifinality).

2.4 The Effect of Prior Strength in the Test Phase

In addition to the simulations of diversity in the developmental learning phase, we investigated the impact of the meta-prior manipulation in the inference process (test phase). This manipulation simulated episodic changes in the neural systems under the normal meta-prior and stable environment conditions, such as hypo- and hyper prior hypothesis of schizophrenia (13). Because synaptic weights were not

updated in the inference process, the increase of meta-priors made the posterior distribution closer to the prior distributions.

In the episodic change simulations, each of the 20 networks trained under normal meta-prior and stable environment conditions executed the flexibility tasks under three prior strength conditions (middle-, hyper-, and hypo-prior conditions). Meta-priors during the flexibility task were set to 0.001, 0.01, and 0.1 for lower, middle, and higher layer under the middle-prior condition, respectively. 10^2 and 10^{-4} times as much meta-priors as middle-prior condition were used in the hyper- and hypo-prior conditions, respectively. As statistical analysis for behavioral and cognitive flexibility, repeated measures, ANOVA, were used (three levels, middle-, hyper-, and hypo-prior conditions).

As in the previous experiment, we quantified two indicators for evaluation of task performance. Behavioral flexibility (Figure S17A), which refers to whether neural networks can accurately predict the observations, declined in the hyper-prior condition than in the hypo- and middle-prior conditions. The middle-prior condition was slightly superior to hypo-prior condition in terms of behavioral flexibility ($F(2, 32) = 289.9875$; $p < 0.0001$ in ANOVA, $t(16) = 19.1019$; $p < 0.0001$ at middle > hyper, $t(16) = 16.3853$; $p < 0.0001$ at hypo > hyper, and $t(16) = 2.9386$; $p = 0.0096$ at middle > hypo).

In contrast, cognitive flexibility (Figure S17B), which refers to whether neural networks can accurately infer true higher-order hidden variables, was improved under the middle-prior conditions than under the hypo-prior conditions ($F(2, 30) = 3.9664$; $p = 0.0296$ in ANOVA, $t(15) = 3.3245$; $p = 0.0139$ at middle > hypo, $t(15) = 0.8651$; $p = 0.4006$ at middle = hyper, $t(15) = 1.6618$; $p = 0.1173$ at hyper = hypo).

Therefore, the decrease in flexibility could be caused not only by anomalies in the developmental learning process but also by altered influences of prior beliefs in the test phase (equifinality). Furthermore, these results suggested that the moderate influence of prior beliefs was important to achieve both accurate prediction of the observed signals (behavioral flexibility) and inference of the higher-order hidden contexts (cognitive flexibility).

SUPPLEMENTARY TABLES

Table S1. Correspondence between generation strategy and metrics.

Procedure	Generation strategy	Target Hierarchy	Metrics Name
Learning	Learning	None	None
Test	Error regression	Observation<br>Latent information	Behavioral flexibility<br>Cognitive flexibility
Analysis	Prior generation<br>Target generation	Observation	Reconstruction ability<br>Learning curve
	Latent space traversal	Latent information	Generative Hierarchy

Table S2. Correspondence between Figure S4 and PV-RNN.

	Figure S4	PV-RNN
Prior model	$p(z_t \| z_{<t})$	$p(z_t \| d_{t-1})$
Posterior model	$q(z_t \| x_{\leq t}, z_{<t})$	$q(z_t \| d_{t-1}, a_t)$
Generation model	$p(x_t \| x_{<t}, z_{\leq t})$	$p(x_t \| d_t)$

Table S3. The results of moderation analysis for generative hierarchy across all layers using multiple regression model. *Note.* Rep: representation efficiently quantified using generative hierarchy in all layers, MP: meta-prior, Env: signal noise levels in environment. Noisy environment condition was coded as "1." * $p < 0.05$, ** $p < 0.01$, *** $p < 0.001$

	Estimate	Std. Error	t value	p value	
(Intercept)	0.8891	0.1173	7.5783	0.0000	***
Rep	-0.2867	0.3472	-0.8259	0.4109	
Env	-0.0912	0.1302	-0.7008	0.4851	
StrongMP	-0.5838	0.1202	-4.8557	0.0000	***
WeakMP	-0.1133	0.1324	-0.8561	0.3941	
Rep x Env	0.3721	0.3799	0.9796	0.3297	
Rep x StrongMP	0.8397	0.3581	2.3449	0.0211	*
Rep x WeakMP	0.3296	0.3851	0.8558	0.3942	
Env x StrongMP	0.5138	0.1512	3.3978	0.0010	**
Env x WeakMP	0.0732	0.1534	0.4768	0.6346	
Rep x Env x StrongMP	-0.8288	0.4373	-1.8952	0.0610	
Rep x Env x WeakMP	-0.3558	0.4411	-0.8067	0.4218	

SUPPLEMENTARY FIGURES

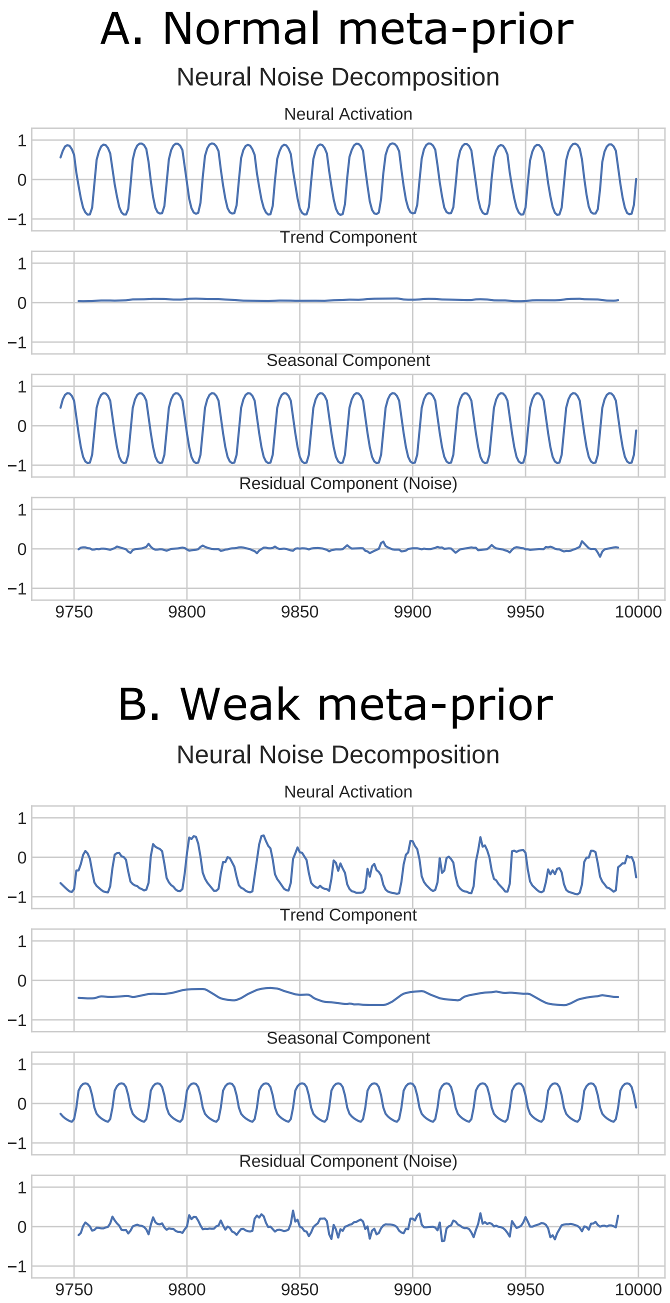

Figure S1. Quantification methods of neural noise using periodic time-series model. The neural activities were decomposed to trend, seasonal, and residual (noise) components. The noise components (the bottom row) under weak meta-prior condition (B) were more than normal meta-prior condition (A).

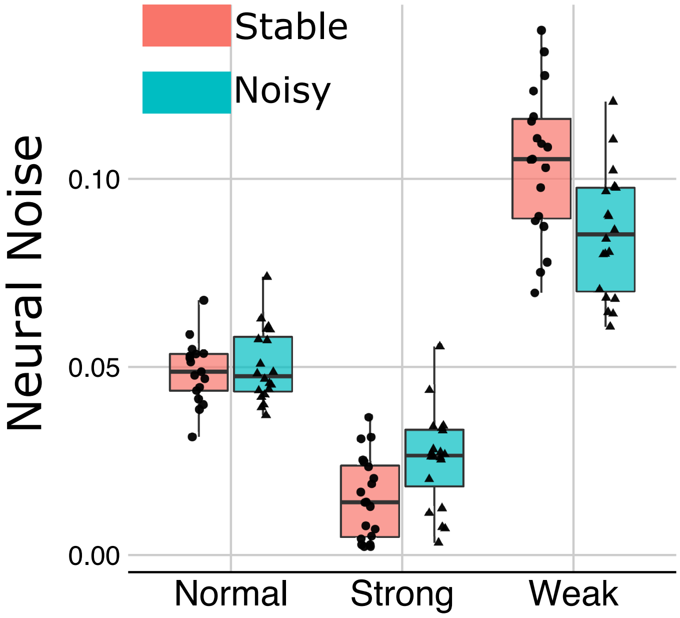

Figure S2. The neural noise in each condition.

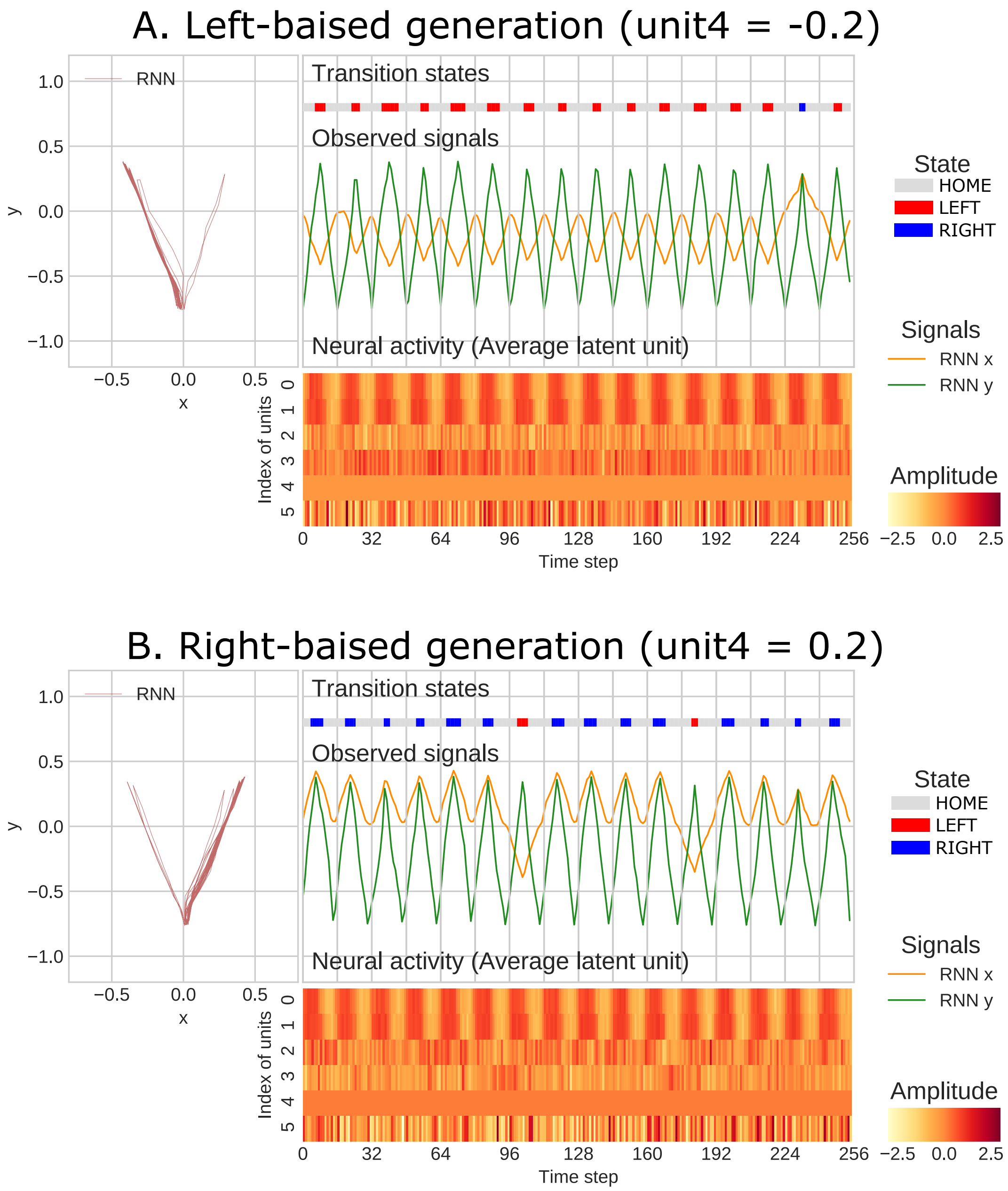

Figure S3. An example of latent space traversal. The outputs and neural activities were generated by a neural network whose unit4 values were fixed at -0.2 (A) and 0.2 (B). This network generated LEFT-biased predictions when unit4 was fixed at -0.2 (A), and RIGHT-biased predictions when unit4 was fixed at 0.2 (B).

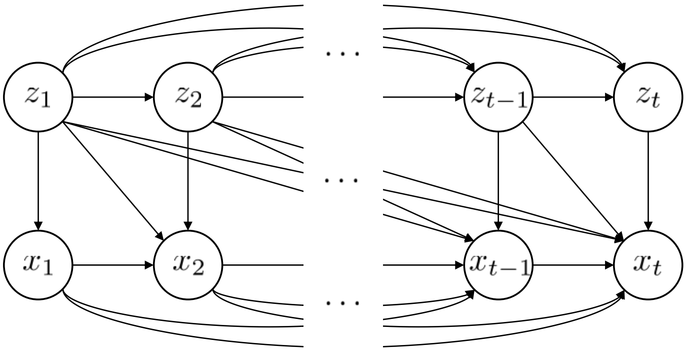

Figure S4. The graphical representation of model used in loss calculation.

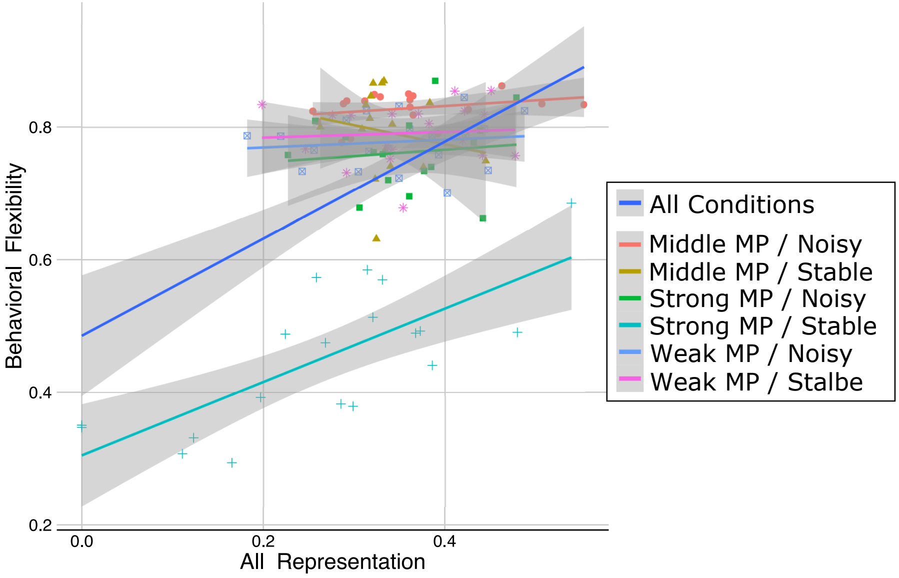

Figure S5. The scatter plot between generative hierarchy across all layers (all representation) and behavioral flexibility.

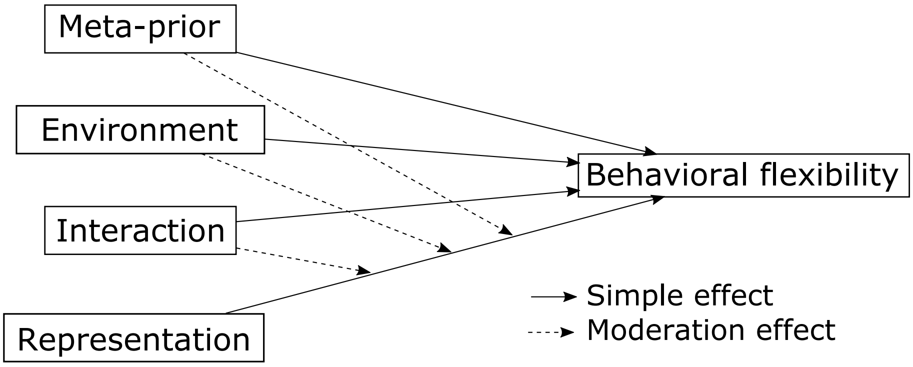

Figure S6. Moderation effect model. “Interaction” in the figure represents the interaction effect term between meta-prior and signal noise level in environment.

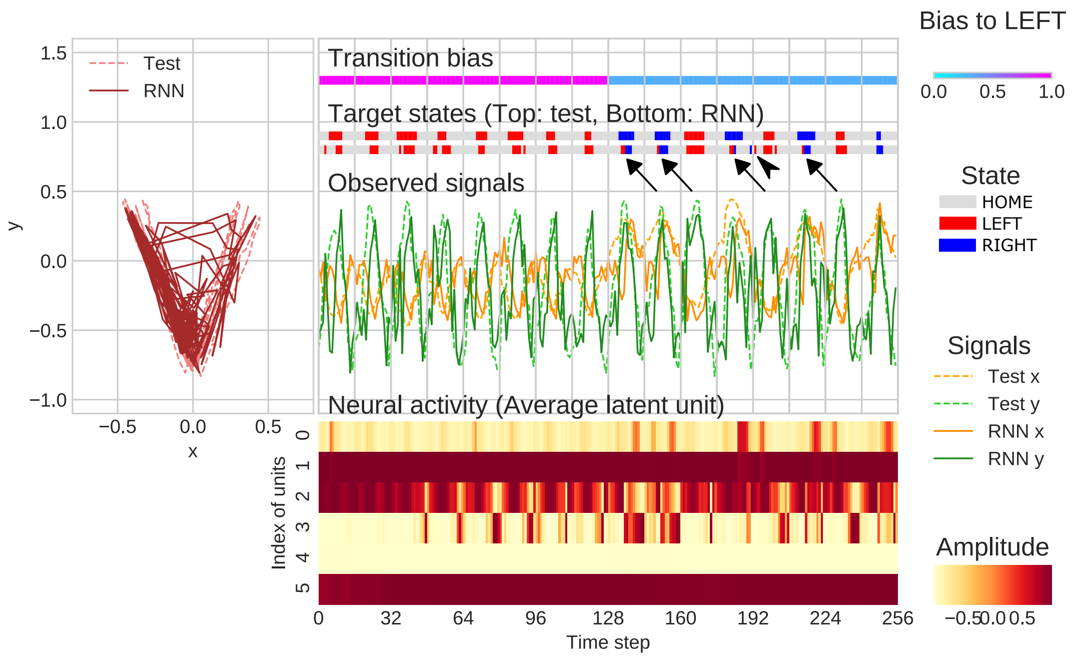

Figure S7. Example of flexibility tasks under long learning condition. The arrows and arrowhead represent preservation error and timing mismatch, respectively. In the figure, only the 128 steps before and after switching of the transition bias are plotted.

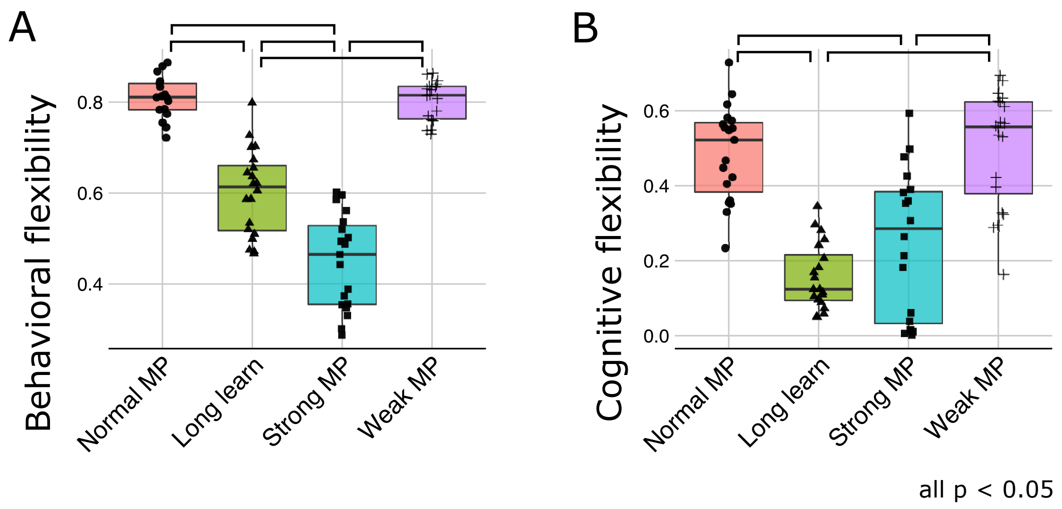

Figure S8. The quantitative evaluation about behavioral (A) and cognitive (B) flexibility including long learning condition.

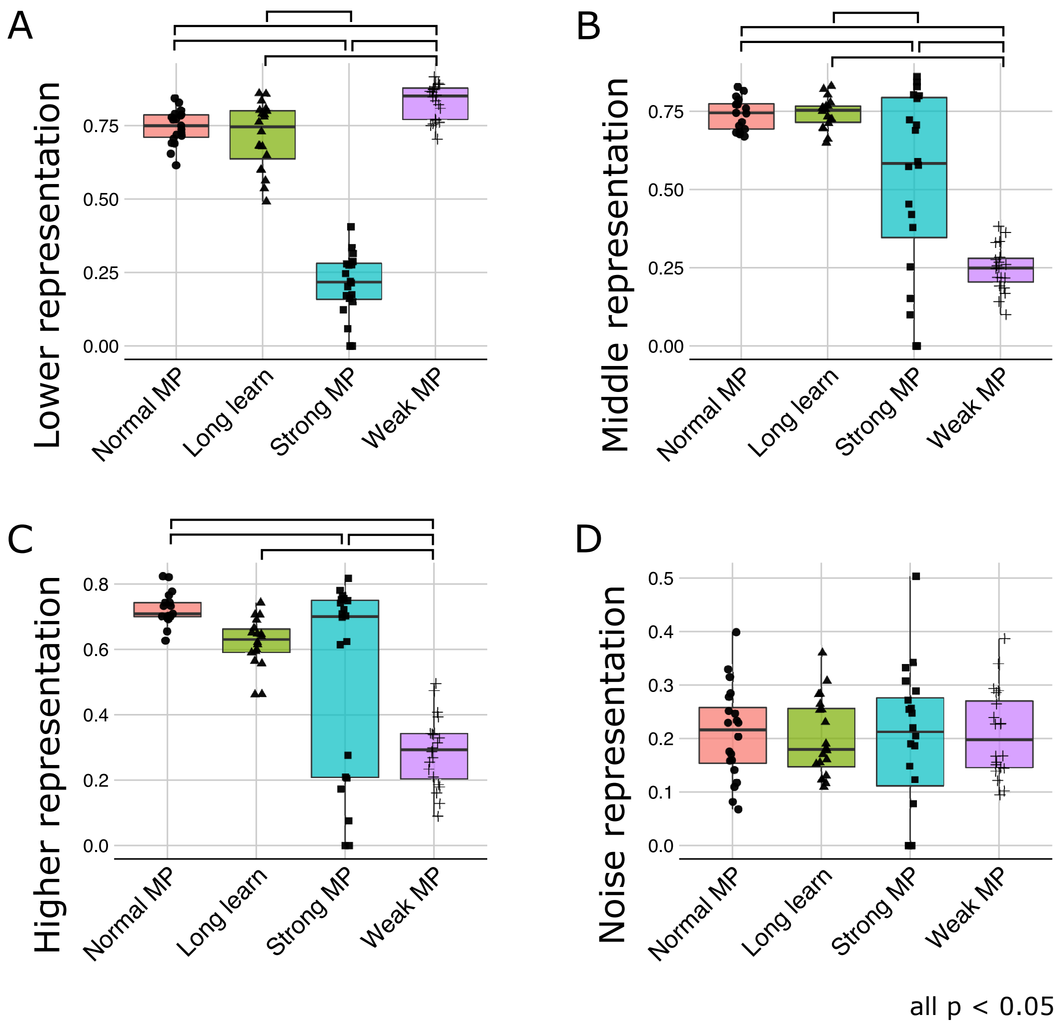

Figure S9. The quantitative evaluation about generative hierarchy including long learning condition.

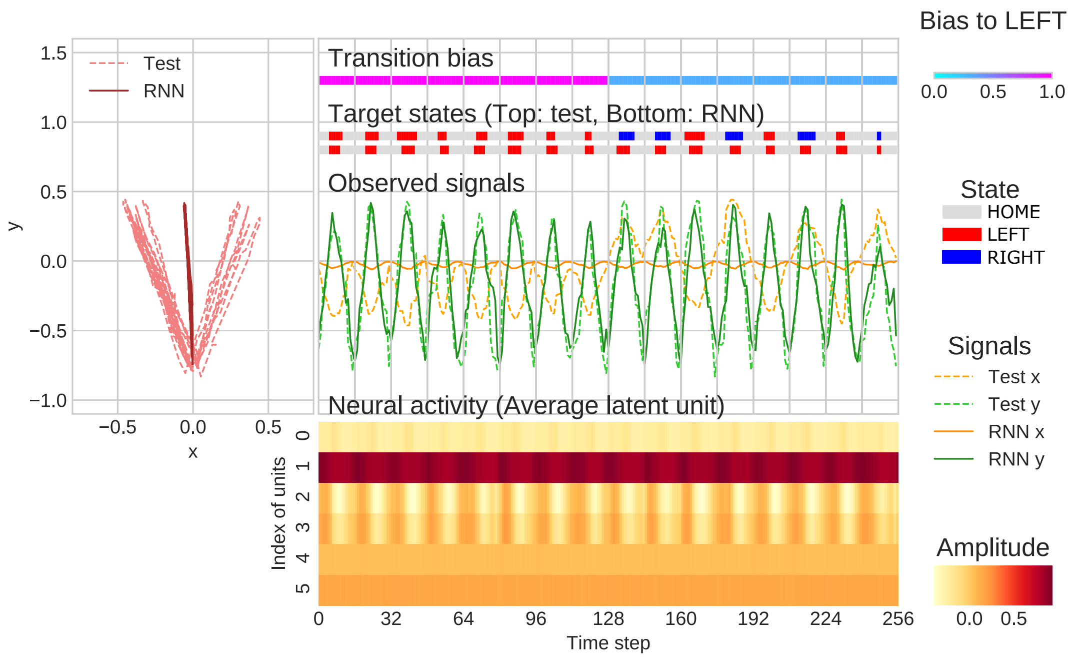

Figure S10. An example of generation pattern, such as restricted and repeated behavior, observed in strong meta-prior condition during the test phase. This network is different from the network presented in the main text. In the figure, only the 128 steps before and after switching of the transition bias are plotted.

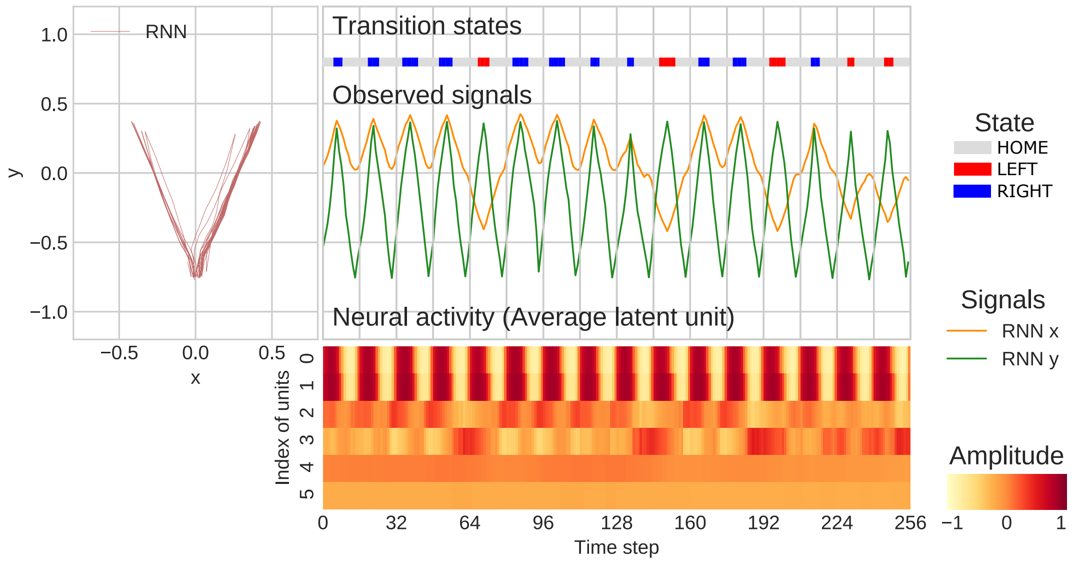

Figure S11. An example of prior generations under normal meta-prior condition. In the figure, the last 256 steps of the sequence with 10,000-steps are plotted.

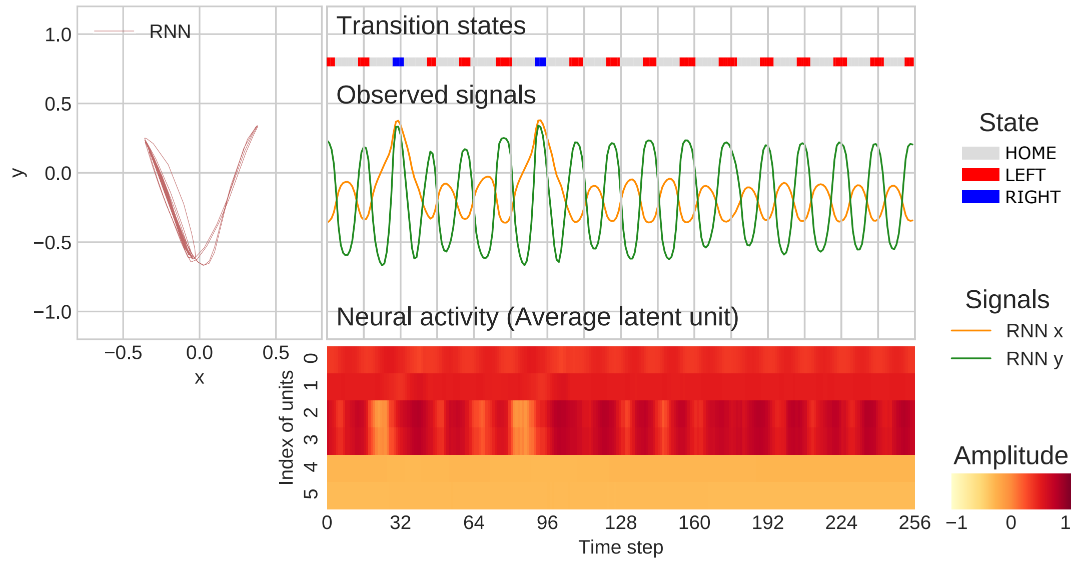

Figure S12. An example of prior generation under strong meta-prior condition. In the figure, the last 256 steps of the sequence with 10,000-steps are plotted.

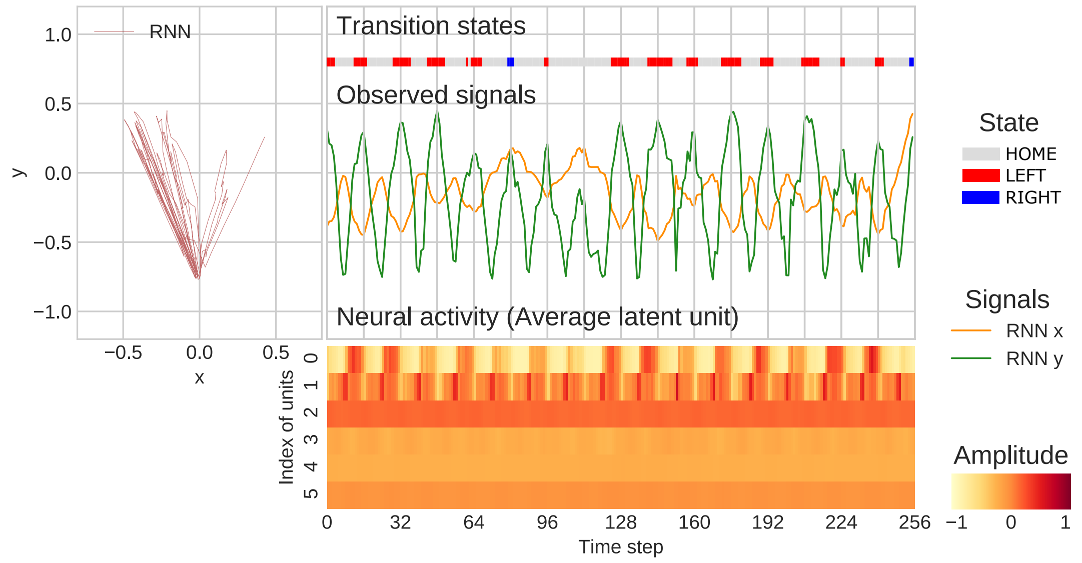

Figure S13. An example of prior generation under weak meta-prior condition. In the figure, the last 256 steps of the sequence with 10,000-steps are plotted.

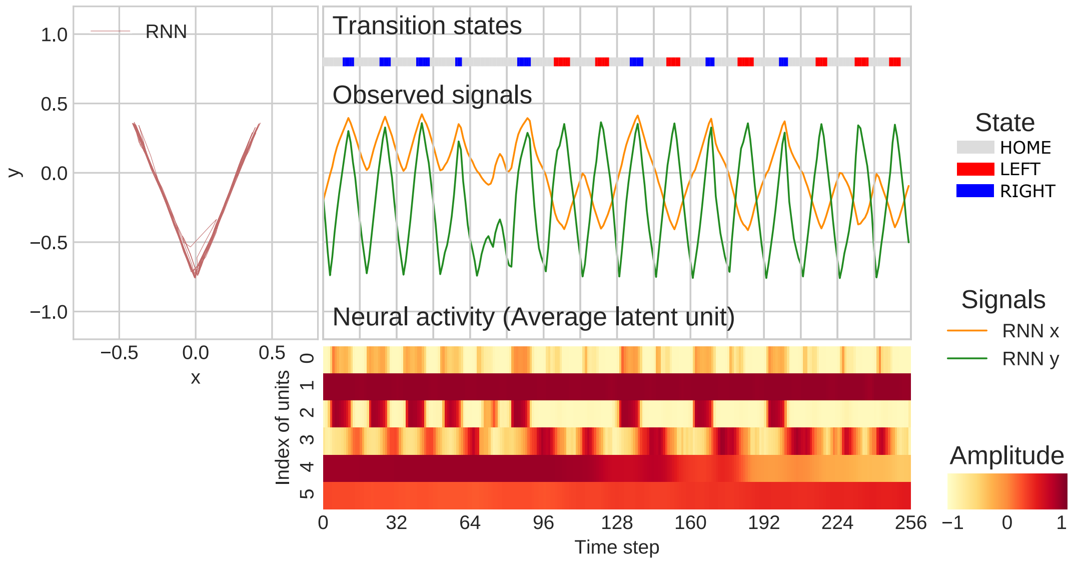

Figure S14. An example of prior generation under long learning condition. In the figure, the last 256 steps of the sequence with 10,000-steps are plotted.

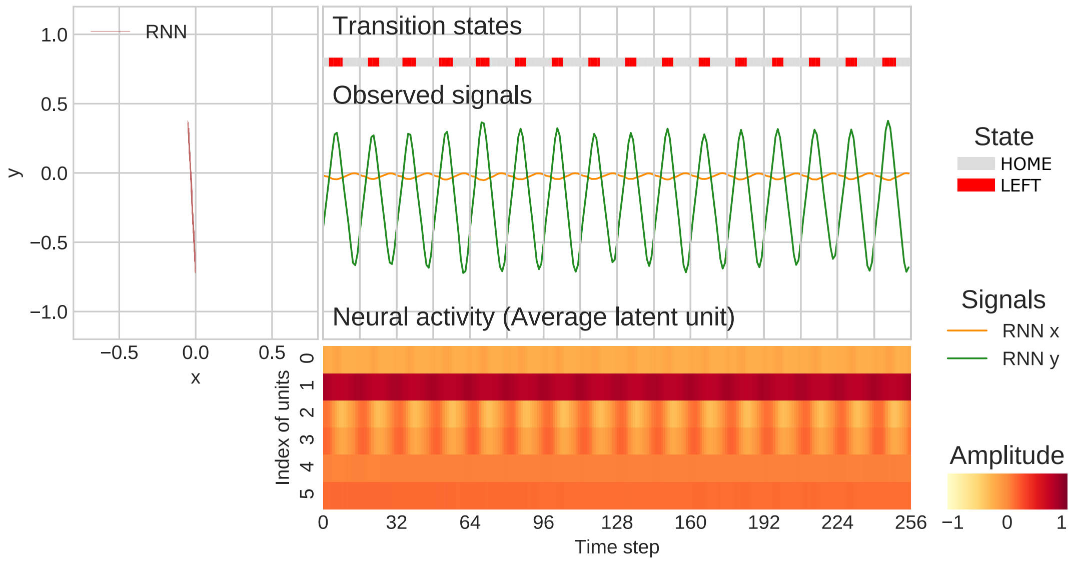

Figure S15. An example of prior generation, such as restricted and repeated behavior, observed in strong meta-prior condition. This network is different from the network presented in the main text. In the figure, the last 256 steps of the sequence with 10,000-steps are plotted.

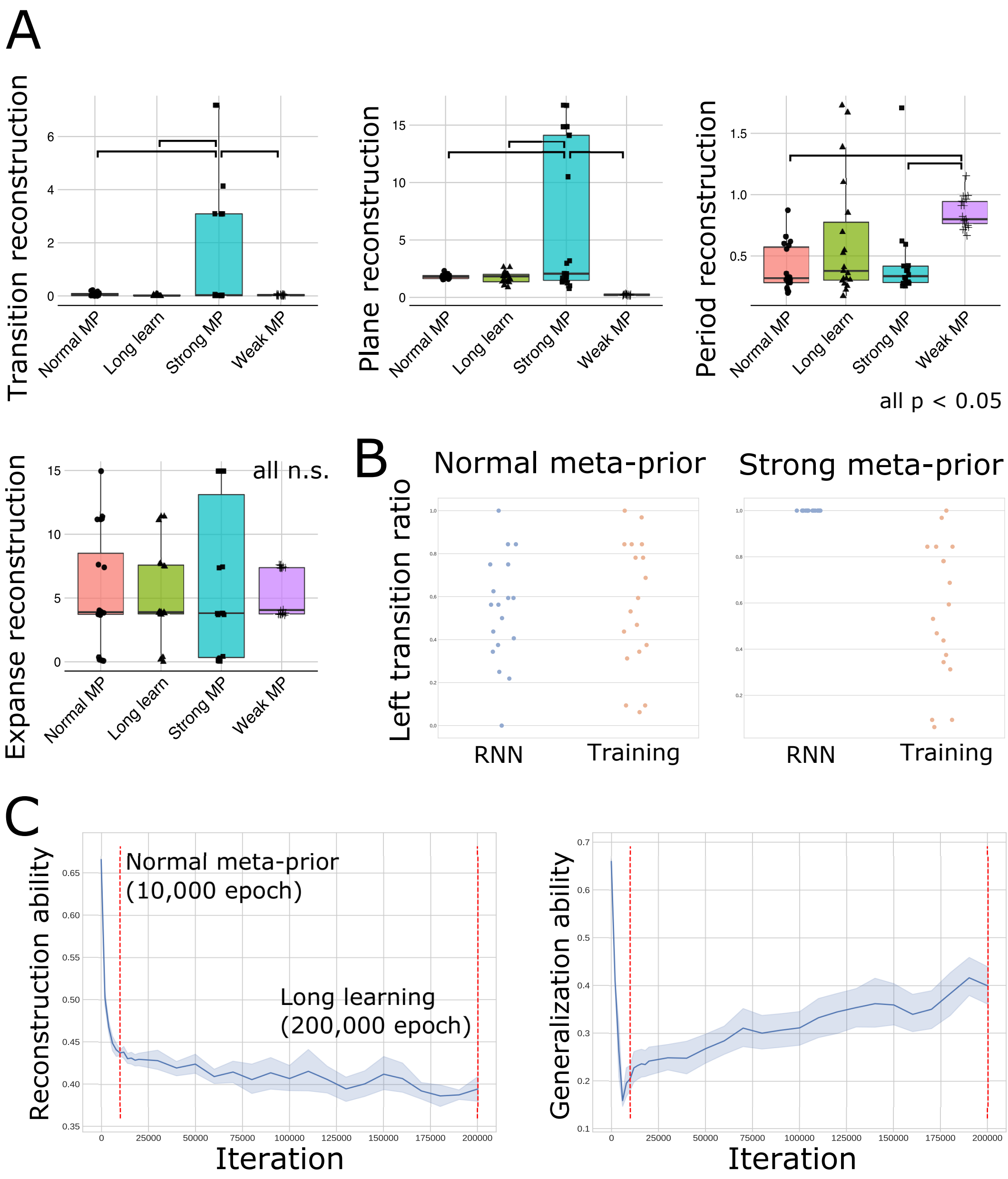

Figure S16. (A) The quantitative analysis for reconstruction abilities. (B) The ratios of LEFT transition in each sequence when prior generations were repeated 18 times under normal (left side) and strong (right side) meta-prior conditions. In this generation, the same networks in each condition were used, but different values were used as random seed during prior generation. The network demonstrating restricted and repeated behavior was used as the network under strong meta-prior condition. (C) The reconstruction and generalization abilities through developmental process (the number of iteration).

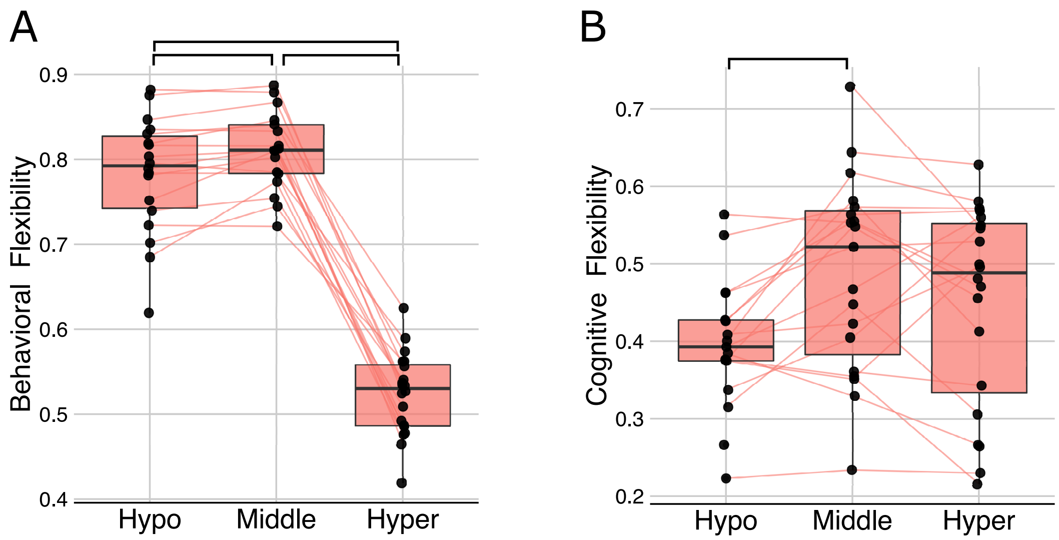

Figure S17. Results of behavioral (A) and cognitive (B) flexibility in manipulating influences of prior belief on posterior belief. The line connecting each prior condition represented individual network.

REFERENCES

1 .Matsumoto T, Tani J. Goal-directed planning for habituated agents by active inference using a variational recurrent neural network. *Entropy* **22** (2020). doi:10.3390/e22050564.

2 .Ohata W, Tani J. Investigation of the sense of agency in social cognition, based on frameworks of predictive coding and active inference: A simulation study on multimodal imitative interaction. *Front Neurorobot* **14** (2020). doi:10.3389/fnbot.2020.00061.

3 .Ahmadi A, Tani J. A novel predictive-coding-inspired variational rnn model for online prediction and recognition. *Neural Comput* **31** (2019) 2025–2074. doi:10.1162/neco_a_01228.

4 .Yamashita Y, Tani J. Emergence of functional hierarchy in a multiple timescale neural network model: a humanoid robot experiment. *PloS Comput Biol* **4** (2008) e1000220. doi:10.1371/journal.pcbi.1000220.

5 .Yamashita Y, Tani J. Spontaneous prediction error generation in schizophrenia. *PLOS ONE* **7** (2012) 1–8. doi:10.1371/journal.pone.0037843.

6 .Kingma DP, Welling M. Auto-encoding variational bayes. *arXiv preprint arXiv:1312.6114* (2013).

7 .Rezende DJ, Mohamed S, Wierstra D. Stochastic backpropagation and approximate inference in deep generative models. *International conference on machine learning* (2014), 1278–1286.

8 .Chung J, Kastner K, Dinh L, Goel K, Courville AC, Bengio Y. A recurrent latent variable model for sequential data. *Advances in neural information processing systems* **28** (2015).

9 .Kingma DP, Ba J. Adam: A method for stochastic optimization. *arXiv preprint arXiv:1412.6980* (2014).

10 .Revelle W. *psych: Procedures for Psychological, Psychometric, and Personality Research*. Northwestern University, Evanston, Illinois (2022). R package version 2.2.3.

11 .R Core Team. *R: A Language and Environment for Statistical Computing*. R Foundation for Statistical Computing, Vienna, Austria (2020).

12 .Bakouie F, Zendehrouh S, Gharibzadeh S. Does a kind of over-fitting occur in the brain of autistic patients? *J Neuropsychiatry Clin Neurosci* **21** (2009) 343–343. doi:10.1176/jnp.2009.21.3.343.

13 .Adams RA, Stephan KE, Brown HR, Frith CD, Friston KJ. The computational anatomy of psychosis. *Front Psychiatry* **4** (2013) 47. doi:10.3389/fpsyt.2013.00047.
